# Familial Risks of Kidney Failure in Sweden: A Nationwide Family Study

**DOI:** 10.1371/journal.pone.0113353

**Published:** 2014-11-25

**Authors:** Delshad Saleh Akrawi, Xinjun Li, Jan Sundquist, Kristina Sundquist, Bengt Zöller

**Affiliations:** 1 Center for Primary Care Research, Lund University/Region Skåne, Malmö, Sweden; 2 Stanford Prevention Research Center, Stanford University School of Medicine, Stanford, California, United States of America; University of Utah School of Medicine, United States of America

## Abstract

**Background:**

The value of family history as a risk factor for kidney failure has not been determined in a nationwide setting.

**Aim:**

This nationwide family study aimed to determine familial risks for kidney failure in Sweden.

**Methods:**

The Swedish multi-generation register on 0–78-year-old subjects were linked to the Swedish patient register and the Cause of death register for 1987–2010. Individuals diagnosed with acute kidney failure (n = 10063), chronic kidney failure (n = 18668), or unspecified kidney failure (n = 3731) were included. Kidney failure patients with cystic kidney disease, congenital kidney and urinary tract malformations, urolithiasis, and rare inherited kidney syndromes, and hyperoxaluria were excluded. Standardized incidence ratios (SIRs) were calculated for individuals whose parents/siblings were diagnosed with kidney failure compared to those whose parents or siblings were not.

**Results:**

The concordant (same disease) familial risks (sibling/parent history) were increased for chronic kidney failure SIR = 2.02 (95% confidence interval, CI 1.90–2.14) but not for acute kidney failure SIR = 1.08 (95% CI 0.94–1.22) and for unspecified kidney failure SIR = 1.25 (95% CI 0.94–1.63). However, the discordant (different disease) familial risk for acute kidney failure SIR = 1.19 (95% CI 1.06–1.32) and unspecified kidney failure SIR = 1.63 (95% CI 1.40–1.90) was significantly increased in individuals with a family history of chronic kidney failure. The familial risk for chronic kidney failure was similar for males SIR = 2.04 (95% CI 1.90–2.20) and females SIR = 1.97 (95% CI 1.78–2.17). Familial risks for chronic kidney failure were highest at age of 10–19 years SIR = 6.33 (95% CI 4.16–9.22).

**Conclusions:**

The present study shows that family history is an important risk factor for chronic kidney failure but to a lower degree for acute kidney failure and unspecified kidney failure.

## Introduction

Chronic kidney disease (CKD) is a worldwide medical problem with poor outcomes, high costs, and increased risk of cardiovascular comorbidities and all-cause mortality [1]–[3]. In developed countries, it is associated with old age, diabetes, hypertension, obesity, and cardiovascular disease [1]. Diabetic glomerulosclerosis and hypertensive nephrosclerosis are the presumed pathological entities but exact diagnosis is often difficult [1]. Familial and genetic factors are increasingly recognized as important for the development of CKD and end-stage renal disease (ESRD) [4]–[8]. Ferguson et al. first reported that family history of ESRD is common among African Americans with ESRD [9]. Several case-series and case-control studies have confirmed the importance of family history of kidney disease in different populations of patients with CKD and/or ERSD [10]–[18]. However, few follow-up studies have determined the importance of family history of CKD and/or ESRD [19]. In one such study, Hsu et al. found a modest effect of self-reported family history of kidney disease (hazard ratio (HR) = 1.40) [19]. No study has determined whether familial factors influence the risk of acute kidney failure, which is an increasing global problem [20].

Though multiple genetic loci have been associated with progressive kidney failure and function, [21]–[23] heritability estimates suggests that only a small proportion of the total heritable contribution to the phenotypic variation of CKD have been identified. Large-scale follow-up studies to determine the importance of family history of CKD may therefore be of clinical value for risk assessment, and may help for planning of genetic studies. Clustering of a disease in families may be caused both by both genetic and non-genetic factors [24]. Increased familial risks may indicate shared environmental and lifestyle factors are of importance for disease development, and not only inherited biological factors [25]. However, without familial clustering a genetic cause is unlikely [24].

To our knowledge, there has not been any nationwide follow-up study whose aim was to determine familial risks of kidney failure among offspring/siblings. This nationwide follow-up study determined the familial risks of different forms of kidney failure – chronic kidney failure, acute kidney failure and unspecified kidney failure (i.e., not specified whether it is acute or chronic) – in the offspring/siblings of individuals with kidney failure. The present study underlines the importance of familial factors in kidney failure.

## Materials and Methods

The dataset used in this study was constructed by linking several national Swedish registers provided by the Swedish government-owned statistics bureau Statistics Sweden and the National Board of Health and Welfare [26]. The Swedish multigenerational register contains information on family relationships for index persons born in Sweden in 1932 and later. Individuals born in 1932 or later and who were alive 1987 constituted the present study population. Linkages were made to National Census data in order to ascertain individual-level socioeconomic status, to the Swedish cause of death register (1987–2010), to the Swedish outpatient care register (2001–2010), and to the Swedish hospital discharge register (1987–2010), the last of which records nationwide dates of hospitalization and hospital diagnoses since 1987. All linkages were performed using the individual national identification number that is assigned to each resident in Sweden for their lifetime. This number was replaced by a serial number in order to preserve anonymity. The serial numbers were used to check that each individual was entered only once (for his or her first main or secondary diagnosis of kidney failure). Over 8.1 million individuals and their biological parents (3.8 million families) were included in the database; the oldest (born in 1932) were 78 years at the end of follow-up period, which ran from 1987–2010.

### Predictor and outcome variables

The predictor variable was family history (in a sibling or parent) of kidney failure (defined below) between 1987 and 2010. Separate risks were also determined for parental and sibling history of kidney failure. The outcome variable was first main or secondary event of kidney failure (acute kidney failure, chronic kidney failure, unspecified kidney failure) in the Swedish hospital discharge register, the Swedish outpatient care register, or the Swedish cause of death register. Acute kidney failure was defined by the following ICD codes: 584 (ICD-9) and N17 (ICD-10). Unspecified kidney failure was defined by the following ICD codes: 586 (ICD-9) and N19 (ICD-10). Chronic kidney failure was defined by the following ICD and surgical codes: 585,V45B, and V56 (ICD-9); N18, N26, T82.4, Y84.1, Z49, Z94.0, and Z99.2 (ICD-10); 6070, 6071, 6072, 6073, 6077, 6079, 9211, 9212, 9213, 9314, and 9200 (dialysis or kidney transplantation related surgical codes for 1987–1996); and V9211, V9212, V9200, V9531, V9532, V9507, KAS00, KAS10, KAS20, KAS40, KAS50, KAS60, KAS96, KAS97, and JAK10, TJA33, TJA35, TKA20 (dialysis or kidney transplantation related procedure and surgical codes for 1997–2010). Only main and secondary diagnoses were considered to ensure high validity. Kidney failure patients with cystic kidney disease (Q61, ICD-10; and 753B, ICD-9), congenital kidney and urinary tract malformations (Q60, Q62, Q63, Q64, ICD-10; and 753A, 753C, 753D, 753E, 753F, 753G, 753H, 753W, 753X, ICD-9), urolithiasis (N20-N23, ICD-10; and 592, ICD-9), rare inherited kidney diseases such as Alports syndrome and Laurence Moon-Biedl-Bardet syndrome (Q87.8A, Q87.8B, ICD-10), and hyperoxaluria (E74.8B, ICD-10; and 271W, ICD-9) were excluded.

### Individual variables included in the analysis

The following variables were included in the analysis: 1) Sex: males or female; 2) Age: Age at diagnosis was categorized into 5-year groups; 3) Time period: The follow-up period was divided into 5-year intervals in order to adjust for changes in incidence rates over time; 4) Socioeconomic status: For both males and females, socioeconomic status was defined by occupation, which was divided into six groups: (1) farmers, (2) blue-collar workers, (3) white-collar workers, (4) professionals, (5) self-employed workers, and (6) others (economically inactive individuals including unemployed individuals and homemakers); 5) Geographic region of residence: To allow adjustment for regional differences in incidence rates, geographic region of residence was divided into three groups: (1) large city, i.e., Stockholm, Gothenburg, or Malmo; (2) Southern Sweden (excluding the large cities, all of which lie in Southern Sweden); and (3) Northern Sweden; and 6) Comorbidity. Comorbidity was defined as a main or secondary diagnosis at follow-up between 1987 and 2010 with the following ICD-codes in the Swedish hospital discharge register or the Swedish outpatient care register: 1) chronic obstructive pulmonary disease (490–496 (ICD-9) and J40–J47 (ICD-10)); 2) obesity (278A and 278B (ICD-9) and E65 and E66 (ICD-10)); 3) alcoholism and alcohol-related liver disease (291, 303, 571A, 571B, 571C, and 571D (ICD-9) and F10 and K70 (ICD-10)); 4) diabetes mellitus (250 (ICD-9) and E10-E14 (ICD-10)); 5) hypertension (401–405 (ICD-9) and I10-I15 (ICD-10)); 6) coronary heart disease (410–414 (ICD-9) and I20-I25 (ICD-10)); 7) heart failure (428 (ICD-9) and I50 (ICD-10)); 8) hyperlipidaemia (272A, 272B, 272C, 272D, and 272E (ICD-9) and E78.0, E78.1, E78.2, E78.3, E78.4, and E78.5 (ICD-10)); and 9) stroke (430–438 (ICD-9) and I60-I69 (ICD-10)).

### Statistical Analysis

For the analysis of familial risks of kidney failure, a previously described method was used [27]. The method is described in detail by Hemminki et al [28] and takes into account clustering within families, since it is based on complete ascertainment of sib ships in affected individuals. Person-years at risk (i.e., the number of persons at risk multiplied by the time at risk) were calculated from the start of the follow-up on 1 January 1987 until diagnosis for kidney failure, death, emigration, or the end of the follow-up (31 December 2010) [29]. Age-adjusted incidence rates were calculated for the whole follow-up period, divided into 5-year periods [29]. Standardized incidence ratios (SIRs) were used to measure the relative risk of kidney failure in individuals with one or more parents with a history of kidney failure compared with individuals with parents without a history of kidney failure. Similar calculations were performed separately for siblings.

The familial SIRs were calculated as the ratio of observed (O) and expected (E) numbers of kidney failure cases using the indirect standardization method:
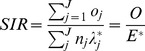
where 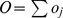 denotes the total observed number of cases in the study group; *E* ^*^ (the expected number of cases) is calculated by applying stratum-specific standard incidence rates (λ^*^_j_) obtained from the reference group to the stratum-specific person-years of risk (*n*_j_) for the study group; *o*_j_ represents the observed number of cases that the cohort subjects contribute to the jth stratum; and J represents the strata defined by cross-classification of the following adjustment variables: age (5-year groups), sex, socioeconomic status, time period (5-year groups), geographic region of residence, and comorbidities. 95% confidence intervals (95% CIs) were calculated assuming a Poisson distribution [29].

Data values are accurate to two decimals places. All analyses were performed using SAS version 9.2 (Institute, Cary, NC, USA).

### Ethical Considerations

Statistics Sweden and the National Board of Health and Welfare maintain the nationwide registers used in the present study. This study was approved by the Ethics Committee at Lund University (approval number 409/2008 Lund with complementary approvals dated September 1, 2009, and January 22, 2010) and recommendations of the Declaration of Helsinki were complied with. The ethics committee waived informed consent as a requirement.

## Results

**Figure 1:**
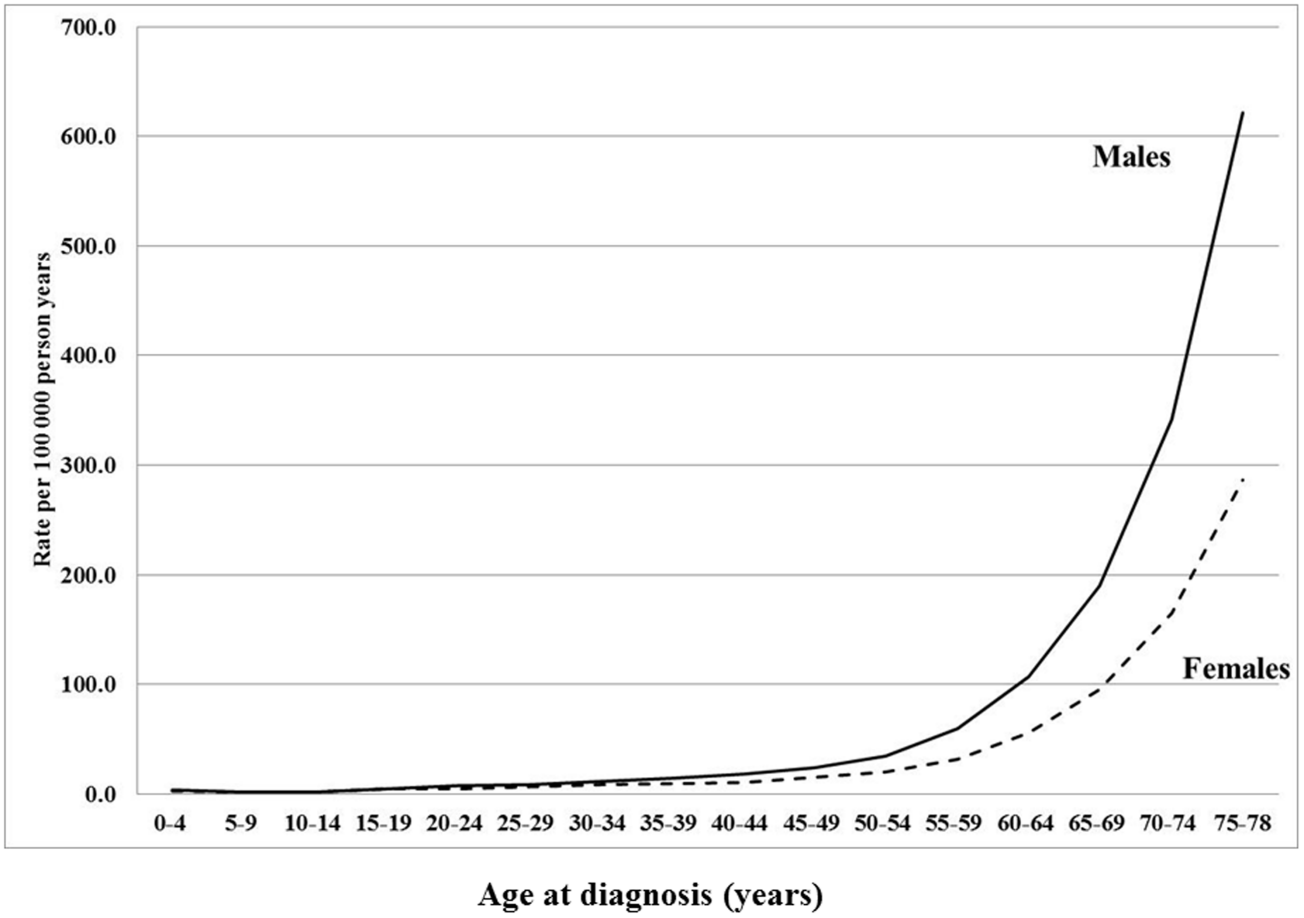
Age-specific incidence rates (per 100000 person years) of kidney failure for males and females in offspring/siblings born in 1932 and later.

**Figure 2:**
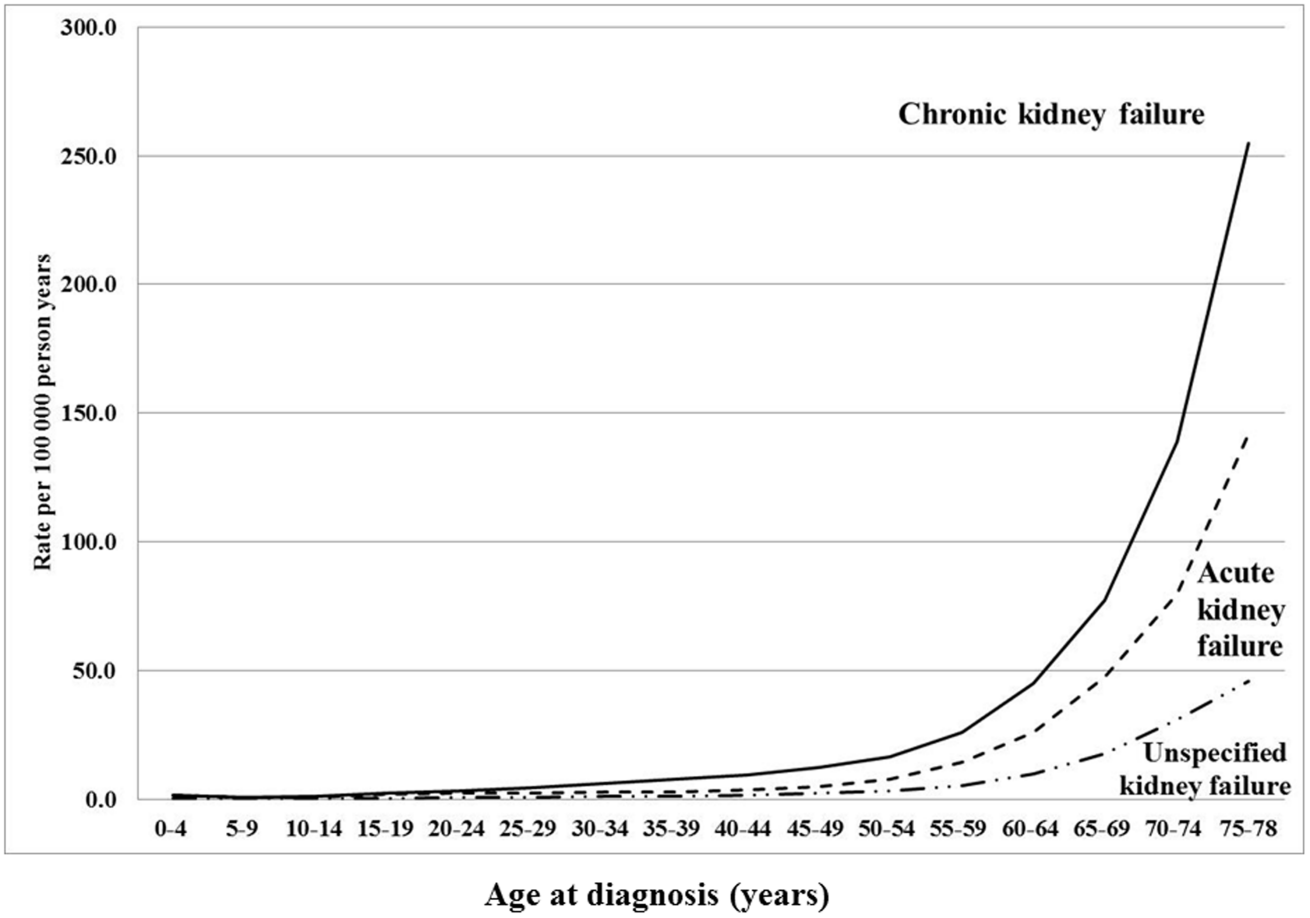
Age-specific incidence rates (per 100000 person years) of chronic kidney failure, acute kidney failure, and unspecified kidney failure ( = others) in offspring/siblings born in 1932 and later.

We analyzed familial risks of kidney failure in the siblings/offspring (aged 0–78 years) of individuals with kidney failure between 1987 and 2010 in Sweden. The population and number of diagnosis for kidney failure are presented in Table 1. A total of 8054071 individuals were included in this cohort. A total of 32462 individuals were diagnosed with kidney failure, 64% (20688) were males and 36% (11774) females (Table 1). Of these patients, 31.0% (10063) were diagnosed with acute kidney failure, 57.5% (18668) with chronic kidney failure, and 11.5% (3731) with unspecified kidney failure. Comorbidities were more common in patients with kidney failure than in the general population (Table 1). The lowest incidence rates for kidney failure were observed for children (Figure 1). The incidence rate for kidney failure increased with age in both sexes (Figure 1). At older ages, the incidence rate for kidney failure was higher for males than females (Figure 1). The incidence rate was highest for chronic kidney failure, and lowest for unspecified kidney failure (Figure 2).

**Table 1:** Study population and number of kidney failure events in individuals aged 0 to 78 years (born 1932 and later and alive in 1987).

	Males				Females				All			
	Population		Kidney failure events		Population		Kidney failure events		Population		Kidney failure events	
	No	%	No	%	No	%	No	%	No	%	No	%
**Age at diagnosis (years)**												
0–9			286	1.4			263	2.2			549	1.7
10–19			424	2.0			419	3.6			843	2.6
20–29			998	4.8			706	6.0			1704	5.2
30–39			1695	8.2			1100	9.3			2795	8.6
40–49			2698	13.0			1585	13.5			4283	13.2
50–59			4712	22.8			2557	21.7			7269	22.4
60–69			6700	32.4			3477	29.5			10177	31.4
70–78			3175	15.3			1667	14.2			4842	14.9
**Subtype of kidney failure**												
Acute kidney failure			6385	30.9			3678	31.2			10063	31.0
Chronic kidney failure			11872	57.4			6796	57.7			18668	57.5
Unspecified kidney failure			2431	11.7			1300	11.1			3731	11.5
**Socioeconomic status**												
Farmer	69645	1.7	609	2.9	50935	1.3	263	2.2	120580	1.5	872	2.7
Self-employed	161705	3.9	1591	7.7	106967	2.7	476	4.0	268672	3.3	2067	6.4
Professional	359536	8.7	2435	11.8	251700	6.4	720	6.1	611236	7.6	3155	9.7
White collar worker	1192177	29.0	5898	28.5	1390397	35.3	4421	37.5	2582574	32.1	10319	31.8
Blue-collar worker	1848695	45.0	9952	48.1	1689070	42.8	5750	48.8	3537765	43.9	15702	48.4
Other	480443	11.7	203	1.0	452801	11.5	144	1.2	933244	11.6	347	1.1
**Region of residence**												
Northern Sweden	427832	10.4	2120	10.2	402535	10.2	1290	11.0	830367	10.3	3410	10.5
Large city	1632588	39.7	8828	42.7	1575746	40.0	4861	41.3	3208334	39.8	13689	42.2
Southern Sweden	2051781	49.9	9740	47.1	1963589	49.8	5623	47.8	4015370	49.9	15363	47.3
**Chronic obstructive pulmonary disease**												
No	3910183	95.1	18979	91.7	3763810	95.5	10467	88.9	7673993	95.3	29446	90.7
Yes	202018	4.9	1709	8.3	178060	4.5	1307	11.1	380078	4.7	3016	9.3
**Obesity**												
No	4080976	99.2	20058	97.0	3885685	98.6	11217	95.3	7966661	98.9	31275	96.3
Yes	31225	0.8	630	3.0	56185	1.4	557	4.7	87410	1.1	1187	3.7
**Alcoholism and related liver disease**												
No	3994406	97.1	18453	89.2	3883021	98.5	11184	95.0	7877427	97.8	29637	91.3
Yes	117795	2.9	2235	10.8	58849	1.5	590	5.0	176644	2.2	2825	8.7
**Diabetes Mellitus**												
No	3998103	97.2	13930	67.3	3868649	98.1	8345	70.9	7866752	97.7	22275	68.6
Yes	114098	2.8	6758	32.7	73221	1.9	3429	29.1	187319	2.3	10187	31.4
**Hyptertension**												
No	3928928	95.5	10841	52.4	3791314	96.2	6965	59.2	7720242	95.9	17806	54.9
Yes	183273	4.5	9847	47.6	150556	3.8	4809	40.8	333829	4.1	14656	45.1
**Coronary heart disease**												
No	3975828	96.7	15160	73.3	3879307	98.4	9561	81.2	7855135	97.5	24721	76.2
Yes	136373	3.3	5528	26.7	62563	1.6	2213	18.8	198936	2.5	7741	23.8
**Stroke**												
No	4038432	98.2	17537	84.8	3892316	98.7	10276	87.3	7930748	98.5	27813	85.7
Yes	73769	1.8	3151	15.2	49554	1.3	1498	12.7	123323	1.5	4649	14.3
**Hyperlipidemia**												
No	4067712	98.9	19437	94.0	3917158	99.4	11233	95.4	7984870	99.1	30670	94.5
Yes	44489	1.1	1251	6.0	24712	0.6	541	4.6	69201	0.9	1792	5.5
**Heart failure**												
No	4068915	98.9	16408	79.3	3920737	99.5	9833	83.5	7989652	99.2	26241	80.8
Yes	43286	1.1	4280	20.7	21133	0.5	1941	16.5	64419	0.8	6221	19.2
All	4112201	100.0	20688	100.0	3941870	100.0	11774	100.0	8054071	100.0	32462	100.0

### Familial risk of kidney failure

**Figure 3:**
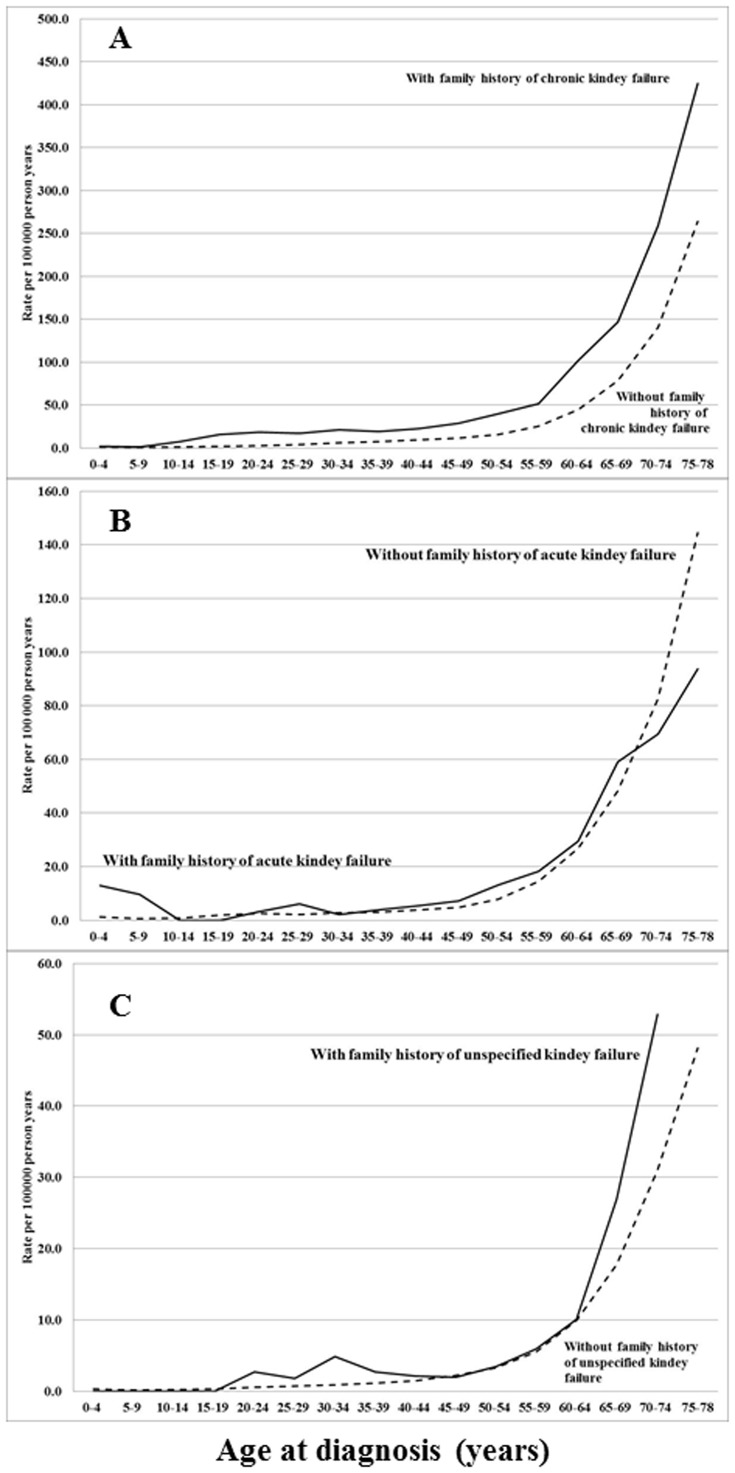
Age-specific incidence rate (per 100000 person years) of kidney failure by concordant family history of kidney failure in individuals born in 1932 and later. A Chronic kidney failure. B Acute kidney failure. C Unspecified kidney failure.

Familial risks of kidney failure according to disease subtypes are presented in Table 2. Familial risks were adjusted for age, sex, time period, region of residence, socioeconomic status, and comorbidities. The incidence rates for familial and non-familial kidney failure are presented Figure 3. Concordant (same disease in proband and exposed relative) and discordant (different disease in proband and exposed relative) risks were determined. The familial risks were highest for chronic kidney failure: the concordant familial SIR for chronic kidney failure was 2.02. The concordant familial risk was not significantly increased for acute kidney failure (SIR = 1.08) and for unspecified kidney failure (SIR = 1.25) (Table 2). However, discordant risks show that family history (sibling/parent) of chronic kidney failure is a risk factor for both acute kidney failure (SIR = 1.19) and unspecific kidney failure (SIR = 1.63) (Table 2). Moreover, discordant risks show that family history (sibling/parent) of acute kidney failure is a risk factor for both chronic kidney failure (SIR = 1.10) and unspecific kidney failure (SIR = 1.30) (Table 2). Family history of unspecified kidney failure (sibling/parent) was a risk factor for chronic kidney failure (SIR = 1.31) (Table 2). Family history of all kidney failure was a risk factor for all types of kidney failure (Table 2). Familial risks of kidney failure were determined in both males and females. There were no major sex differences (Table 2).

**Table 2:** Familial concordant and discordant risk (sibling/parent history) of kidney failure in males and females.

		Males				Females				All			
Type of kidney failure in proband	Subtype of kidney failure in offspring/sibling	O	SIR	95% CI		O	SIR	95% CI		O	SIR	95% CI	
Acute kidney failure	Acute kidney failure	153	1.09	0.92	1.27	84	1.05	0.84	1.30	237	1.08	0.94	1.22
	Chronic kidney failure	282	1.07	0.95	1.20	153	1.15	0.98	1.35	435	**1.10**	**1.00**	**1.21**
	Unspecified kidney failure	64	**1.29**	**1.00**	**1.65**	36	1.31	0.92	1.81	100	**1.30**	**1.06**	**1.58**
	All kidney failure	499	**1.10**	**1.01**	**1.20**	273	**1.14**	**1.01**	**1.28**	772	**1.11**	**1.04**	**1.19**
Chronic kidney failure	Acute kidney failure	201	1.15	0.99	1.32	129	1.26	1.05	1.50	330	**1.19**	**1.06**	**1.32**
	Chronic kidney failure	717	**2.04**	**1.90**	**2.20**	395	**1.97**	**1.78**	**2.17**	1112	**2.02**	**1.90**	**2.14**
	Unspecified kidney failure	104	**1.56**	**1.28**	**1.89**	65	**1.76**	**1.36**	**2.24**	169	**1.63**	**1.40**	**1.90**
	All kidney failure	1022	**1.72**	**1.62**	**1.83**	589	**1.73**	**1.59**	**1.88**	1611	**1.73**	**1.64**	**1.81**
Unspecified kidney failure	Acute kidney failure	72	1.07	0.84	1.35	42	1.04	0.75	1.41	114	1.06	0.88	1.28
	Chronic kidney failure	176	**1.31**	**1.12**	**1.52**	101	**1.31**	**1.07**	**1.60**	277	**1.31**	**1.16**	**1.47**
	Unspecified kidney failure	33	1.18	0.81	1.65	21	1.38	0.85	2.11	54	1.25	0.94	1.63
	All kidney failure	281	**1.22**	**1.09**	**1.38**	164	**1.24**	**1.06**	**1.44**	445	**1.23**	**1.12**	**1.35**
All kidney failure	Acute kidney failure	426	**1.11**	**1.01**	**1.22**	255	**1.15**	**1.01**	**1.30**	681	**1.12**	**1.04**	**1.21**
	Chronic kidney failure	1175	**1.57**	**1.48**	**1.66**	649	**1.58**	**1.46**	**1.71**	1824	**1.57**	**1.50**	**1.65**
	Unspecified kidney failure	201	**1.39**	**1.21**	**1.60**	122	**1.53**	**1.27**	**1.83**	323	**1.44**	**1.29**	**1.61**
	All kidney failure	1802	**1.41**	**1.35**	**1.48**	1026	**1.44**	**1.35**	**1.53**	2828	**1.42**	**1.37**	**1.48**

Familial risks were adjusted for age, sex, time period, region of residence, socioeconomic status, and comorbidities.

Bold type: 95% CI does not include 1.00.

O = observed number of cases with family history of kidney failure; SIR = standardized incidence ratio; CI = confidence interval.

In Table 3, familial concordant risks are presented according to the affected relative. Familial risks were adjusted for age, sex, time period, region of residence, socioeconomic status, and comorbidities. Sibling history of chronic kidney failure showed the highest familial risk, with a concordant SIR of 2.52. The familial concordant risk for individuals with a parental history of chronic kidney failure was 1.67. There were no major sex differences. The familial concordant risks for acute and unspecified kidney failure were not significant (Table 3).

**Table 3:** Familial risk of concordant kidney failure in males and females.

		Males				Females				All			
Probands with any type of kidney failure	Subtype of kidney failure in offspring/siblings	O	SIR	95% CI		O	SIR	95% CI		O	SIR	95% CI	
Family history (parent/sibling)	Acute kidney failure	153	1.09	0.92	1.27	84	1.05	0.84	1.30	237	1.08	0.94	1.22
	Chronic kidney failure	717	**2.04**	**1.90**	**2.20**	395	**1.97**	**1.78**	**2.17**	1112	**2.02**	**1.90**	**2.14**
	Unspecified kidney failure	33	1.18	0.81	1.65	21	1.38	0.85	2.11	54	1.25	0.94	1.63
	All kidney failure	1802	**1.41**	**1.35**	**1.48**	1026	**1.44**	**1.35**	**1.53**	2828	**1.42**	**1.37**	**1.48**
Parents history	Acute kidney failure	103	1.06	0.87	1.29	58	1.10	0.83	1.42	161	1.07	0.91	1.25
	Chronic kidney failure	378	**1.71**	**1.54**	**1.89**	204	**1.62**	**1.40**	**1.85**	582	**1.67**	**1.54**	**1.82**
	Unspecified kidney failure	22	1.00	0.63	1.52	16	1.50	0.86	2.45	38	1.16	0.82	1.60
	All kidney failure	1124	**1.28**	**1.21**	**1.36**	637	**1.32**	**1.22**	**1.43**	1761	**1.29**	**1.23**	**1.35**
Paternal history	Acute kidney failure	62	1.20	0.92	1.54	26	1.03	0.67	1.51	88	1.14	0.92	1.41
	Chronic kidney failure	212	**1.73**	**1.50**	**1.98**	105	**1.45**	**1.19**	**1.76**	317	**1.62**	**1.45**	**1.81**
	Unspecified kidney failure	9	0.74	0.33	1.40	11	1.87	0.93	3.36	20	1.10	0.67	1.71
	All kidney failure	637	**1.35**	**1.24**	**1.45**	326	**1.21**	**1.08**	**1.35**	963	**1.29**	**1.21**	**1.38**
Maternal history	Acute kidney failure	42	0.92	0.66	1.25	33	1.19	0.82	1.67	75	1.02	0.80	1.28
	Chronic kidney failure	178	**1.69**	**1.45**	**1.96**	108	**1.88**	**1.54**	**2.27**	286	**1.76**	**1.56**	**1.97**
	Unspecified kidney failure	13	1.26	0.67	2.16	7	1.39	0.55	2.89	20	1.31	0.80	2.02
	All kidney failure	513	**1.20**	**1.10**	**1.31**	326	**1.47**	**1.31**	**1.63**	839	**1.29**	**1.21**	**1.38**
Sibling history	Acute kidney failure	52	1.15	0.86	1.50	27	0.98	0.64	1.43	79	1.08	0.86	1.35
	Chronic kidney failure	366	**2.52**	**2.27**	**2.80**	213	**2.52**	**2.19**	**2.88**	579	**2.52**	**2.32**	**2.73**
	Unspecified kidney failure	12	1.65	0.85	2.89	6	1.08	0.39	2.37	18	1.40	0.83	2.22
	All kidney failure	738	**1.68**	**1.56**	**1.80**	430	**1.70**	**1.54**	**1.87**	1168	**1.69**	**1.59**	**1.78**

Familial risks were adjusted for age, sex, time period, region of residence, socioeconomic status, and comorbidities.

Bold type: 95% CI does not include 1.00.

O = observed number of cases with family history of kidney failure; SIR = standardized incidence ratio; CI = confidence interval.

The familial concordant risks (parent/sibling history) were stratified according to age at diagnosis (Table 4). The familial risks for chronic kidney failure were highly age dependent and were highest risks at younger ages (SIR = 6.33 between the age of 10 and 19 years). Increased concordant familial risk of 1.81 was noted also for chronic kidney failure for those aged 60 years or more (Table 4). The familial concordant risks for chronic kidney failure were increased in all age groups except those younger than 10 years. For acute kidney failure, the familial concordant risks were only significantly increased only in two age groups (Table 4). The familial risk for acute kidney failure before age of 10 years was high (SIR = 14.21). The age of these six children with familial acute kidney failure were 0, 1, 1, 5, 5, and 7 years. For three children, the diagnosis was unknown (two had ICD diagnosis = Z038 and one had no additional diagnosis). One child was prematurely born (<28 weeks) and/or had a very low birth weight (<1000 g) (ICD-9 = 765A), one had unspecified infectious gastroenteritis (ICD-9 = 009B), and one had gastroenteritis with Escherichia coli (ICD-9 = 008A). No significant increased risk for unspecified kidney failure was observed for any other age groups. However, the familial risk for all kidney failure was increased in all age groups (Table 4).

**Table 4:** Familial risk (sibling/parent history) of concordant kidney failure in males and females by age diagnosis.

	Males				Females				All			
Age at diagnosis (years)	O	SIR	95% CI		O	SIR	95% CI		O	SIR	95% CI	
Acute kidney failure												
<10	4	**16.78**	**4.36**	**43.39**	2	**10.88**	**1.03**	**40.02**	6	**14.21**	**5.11**	**31.14**
10–19	0				0				0			
20–29	6	2.00	0.72	4.37	5	**3.70**	**1.17**	**8.70**	11	**2.52**	**1.25**	**4.53**
30–39	11	1.43	0.71	2.57	2	0.61	0.06	2.24	13	1.18	0.63	2.03
40–49	22	1.23	0.77	1.87	14	1.57	0.86	2.64	36	1.34	0.94	1.86
50–59	52	**1.35**	**1.01**	**1.78**	21	0.84	0.52	1.28	73	1.15	0.90	1.45
> = 60	58	0.80	0.61	1.03	40	0.99	0.71	1.35	98	0.87	0.70	1.06
All	153	1.09	0.92	1.27	84	1.05	0.84	1.30	237	1.08	0.94	1.22
Chronic kidney failure
<10	2	3.92	0.37	14.41	0				2	2.09	0.20	7.70
10–19	16	**6.94**	**3.96**	**11.30**	11	**5.60**	**2.78**	**10.06**	27	**6.33**	**4.16**	**9.22**
20–29	41	**4.87**	**3.49**	**6.61**	33	**4.35**	**2.99**	**6.12**	74	**4.62**	**3.63**	**5.81**
30–39	91	**2.36**	**1.90**	**2.90**	43	**1.76**	**1.27**	**2.37**	134	**2.13**	**1.78**	**2.52**
40–49	135	**2.08**	**1.75**	**2.47**	75	**2.11**	**1.66**	**2.65**	210	**2.09**	**1.82**	**2.40**
50–59	187	**1.86**	**1.60**	**2.14**	92	**1.74**	**1.40**	**2.14**	279	**1.82**	**1.61**	**2.04**
> = 60	245	**1.81**	**1.59**	**2.05**	141	**1.81**	**1.52**	**2.14**	386	**1.81**	**1.63**	**2.00**
All	717	**2.04**	**1.90**	**2.20**	395	**1.97**	**1.78**	**2.17**	1112	**2.02**	**1.90**	**2.14**
Unspecified kidney failure
<10	0				0				0			
10–19	0				0				0			
20–29	1	2.39	0.00	13.67	1	4.41	0.00	25.26	2	3.10	0.29	11.38
30–39	6	**2.80**	**1.01**	**6.12**	1	1.04	0.00	5.95	7	2.25	0.89	4.66
40–49	2	0.58	0.05	2.12	4	1.62	0.42	4.18	6	1.01	0.36	2.21
50–59	8	1.04	0.45	2.07	4	1.18	0.31	3.05	12	1.09	0.56	1.90
> = 60	16	1.13	0.64	1.83	11	1.35	0.67	2.43	27	1.21	0.80	1.76
All	33	1.18	0.81	1.65	21	1.38	0.85	2.11	54	1.25	0.94	1.63
All kidney failure												
<10	6	**3.28**	**1.18**	**7.19**	6	**3.52**	**1.27**	**7.71**	12	**3.40**	**1.75**	**5.95**
10–19	23	**3.43**	**2.17**	**5.15**	24	**4.02**	**2.57**	**5.98**	47	**3.70**	**2.72**	**4.93**
20–29	72	**2.53**	**1.98**	**3.18**	56	**2.83**	**2.14**	**3.68**	128	**2.65**	**2.21**	**3.15**
30–39	180	**1.74**	**1.49**	**2.01**	87	**1.47**	**1.18**	**1.82**	267	**1.64**	**1.45**	**1.85**
40–49	290	**1.45**	**1.29**	**1.63**	178	**1.58**	**1.36**	**1.83**	468	**1.50**	**1.37**	**1.64**
50–59	515	**1.41**	**1.29**	**1.54**	260	**1.30**	**1.15**	**1.47**	775	**1.37**	**1.28**	**1.47**
> = 60	716	**1.25**	**1.16**	**1.35**	415	**1.32**	**1.20**	**1.46**	1131	**1.28**	**1.20**	**1.35**
All	1802	**1.41**	**1.35**	**1.48**	1026	**1.44**	**1.35**	**1.53**	2828	**1.42**	**1.37**	**1.48**

Familial risks were adjusted for age, sex, time period, region of residence, socioeconomic status, and comorbidities.

Bold type: 95% CI does not include 1.00. O = observed number of cases with family history of kidney failure; SIR = standardized incidence ratio; CI = confidence interval.

### Test for the extent of the shared non-genetic familial contribution

In order to test for the extent of environmental sharing in the observed risks of kidney failure SIRs for siblings according to difference in age were calculated (Table S1). Familial risks were adjusted for age, sex, time period, region of residence, socioeconomic status, and comorbidities. Overall, the age difference had little effect. Siblings with an age difference of <5 years showed a SIR for all kidney failure of 1.64 (95% CI, 1.50 to 1.79) compared with 1.72 (95% CI, 1.59 to 1.86) for those with an age difference of ≥5 years. The concordant sibling risk for chronic kidney failure was 2.36 (95% CI 2.07–2.67) for siblings with an age difference of <5 years, compared with 2.65 (95% CI, 2.38 to 2.95) for those with an age difference of ≥5 years.

### Additional analyses

In Table S2, familial concordant and discordant risks are presented according to the affected relative. Familial risks were adjusted for age, sex, time period, region of residence, socioeconomic status, and comorbidities. The results were basically similar to the familial concordant/discordant risk in Table 2. Thus, concordant and discordant risk was generally highest for chronic kidney failure, followed by unspecified kidney failure, and weakest for acute kidney failure independent of the type of affected relative (sibling/parent, parents, mother, father or sibling).

Table S3 shows age stratified concordant and discordant familial risks (parent/siblings) of kidney failure. Familial risks were adjusted for age, sex, time period, region of residence, socioeconomic status, and comorbidities. The results were basically similar to the familial age stratified concordant risks in Table 4. Thus, age stratified concordant and discordant risks were generally highest for chronic kidney failure, followed by unspecified kidney failure, and weakest for acute kidney failure independent of the type of affected relative (sibling/parent, parents, mother, father or sibling). However, for acute kidney failure, the familial concordant risks were highly increased in the two youngest age groups (Table S3).

### Sensitivity analysis

Table S4 presents concordant and discordant familial risks (parent/siblings) after exclusion of patients with kidney cancer in parents/offspring. This did not change the results to any major degree.

Table S5 shows concordant and discordant familial risks (parent/siblings) for the follow up period 2001–2010. The familial risks were similar compared to using a follow-up period from 1987–2010. Thus, inclusion of outpatients with kidney failure diagnosis from 2001 until 2010 did not change the results to any major degree.

## Discussion

The present study is the first nationwide follow-up study to evaluate the familial risks of chronic, acute, and unspecified kidney failure among offspring/siblings of affected individuals. The results confirm previous case-series and case-control studies, which showed that familial factors are important for chronic kidney failure [9]–[18]. The present study adds follow-up data for a whole country. Previously a follow-up study only showed moderately increased familial risk (HR = 1.40) [19]. The present results indicate that familial factors are important for chronic kidney failure in both males and females of all ages (except <10 years), although the familial risks were highest at ages 10–19 years (Table 4). This is in contrast to the findings that most risk alleles from genome wide association studies add little to the prediction of CKD [21]–[23]. It is possible that there are a large number of risk alleles that have yet to be discovered that may account for this discrepancy. Unique familial (environmental or genetic) factors may predispose individuals to chronic kidney failure. Support for a genetic contribution to chronic kidney failure comes from the observation that age difference between siblings had little influence (Table S1). If environmental factors were strong, one would expect higher risks for siblings with smaller age differences. For chronic kidney failure, the familial concordant risks were high (Table 2). The familial concordant risks for acute and unspecified kidney failure were not significant (Table 2). However, increased discordant familial risks show that familial factors also are involved in acute and unspecified kidney failure (Table 2), though to a lower degree than for chronic kidney failure. Acute kidney failure is instead more related to precipitating factors such as sepsis, complex surgery, diagnostic procedures requiring intravenous contrast continue, and drug-induced kidney injury [20]. Unspecified kidney failure is probably a mixture of patients with chronic and acute kidney failure. No previous study has reported familial risks for acute and unspecified kidney failure.

Of interest is the high familial risk for acute renal failure among children younger than ten years (SIR = 14.21) and also children and teenagers between 10 and 19 years of age (SIR = 2.52) (Table 4). Among the children younger than 10 years, 2 were related to infection and one to preterm birth and or/low birth weight, which argue against a genetic cause for these cases of acute renal failure. Three cases were unknown and we cannot exclude that in rare cases familial factors are important among children and teenagers. A study from Norway of acute renal failure identified 315 cases of acute renal failure among children under the age of 16 years [30]. The estimated incidence rate was 3.3 cases per 100 000 children. This is in range of out overall kidney failure incidence rate among children (Table 1). Most cases (43%) in the Norwegian study were children under the age of five years [30]. The authors identified 53 aetiologies and classified these into 30 aetiological groups: 25% were prerenal failure (n = 75), 74% were intrinsic/renal failure (n = 234), and 2% were postrenal failure (n = 5). Nephritic syndromes was the most common cause (44%) of acute kidney failure, followed by haemolytic-uraemic syndrome (HUS) (15%) [30].

The present design has potential advantages and disadvantages. Strengths of the study include complete nationwide coverage from 1987 in a country with high standards of diagnosis, with diagnoses often being made by specialists during extended examinations in clinics. The Swedish hospital discharge register contains no information about diagnostic procedures, which is a limitation. Moreover, the validity of ICD codes for kidney disease has not been reported. However, the Swedish hospital discharge register has been extensively validated and its overall diagnostic validity is close to 90% [31]–[32]. A limitation is the inclusion of asymptomatic early stages of renal failure. The Swedish ICD-9 code 585 has no sub codes for different stages of ESRD. Thus, a number of patients with unidentified early stages of renal failure are not included in the study, which most likely is a non-differential bias with regards to familial risks. Another likely non-differential bias regarding familial risks is that cases in probands and relatives before 1987 are unknown. Moreover, the number of comorbidities is rather low (Table 1), possible due to that diagnosis made in primary health are not included. No nationwide primary health care register exists in Sweden.

Another important strength of our study is that it was based on nationwide registers and was thus free of selection and recall bias. The Swedish multi-generation register and the Swedish hospital discharge register are validated data sources that have been proven to be reliable in the study of many diseases [26], [31]. Data in our dataset are almost 100% complete [26].

In summary, the present study found indications of strong aggregation of chronic kidney failure, while familial factors are less important in acute and unspecified kidney failure. Familial non-genetic factors contribute among husbands but not wives. Identification of the unique familial factors in chronic kidney failure will advance our knowledge about the pathogenesis of kidney failure.

## Supporting Information

Table S1 Familial risk of concordant kidney failure among siblings by age at difference in siblings.
(DOCX)

Table S2 Familial risk of concordant and discordant kidney failure in men and women.
(DOCX)

Table S3 Familial risk (sibling/parent history) of concordant and discordant kidney failure in males and females by age at diagnosis.
(DOCX)

Table S4 Familial risk (sibling/offspring) of concordant and discordant kidney failure in males and females, after excluding kidney cancer in parents/offspring.
(DOCX)

Table S5 Familial risk of concordant and discordant kidney failure in males and females, follow-up 2001–2010.
(DOCX)
