# Supplementary material for: Familial Risks of Kidney Failure in Sweden: A Nationwide Family Study
Source: PLoS One. 2014 Nov 25;9(11):e113353. doi: 10.1371/journal.pone.0113353 (PMC4244139; doi:10.1371/journal.pone.0113353)
Supplement: Table S1 — Familial risk of concordant kidney failure among siblings by age at difference in siblings. (DOCX) [file pone.0113353.s001.docx]

| **Table S1. Familial risk of concordant kidney failure among siblings by age at difference in siblings** | | | | | | |
| --- | --- | --- | --- | --- | --- | --- |
|  | Age difference < 5 years | | | |  |  |
| Subtype of kidney failure in siblings | O | SIR | 95% CI | |  |  |
| Acute kidney failure | 34 | 1.26 | 0.87 | 1.77 |  |  |
| Chronic kidney failure | 242 | **2.36** | **2.07** | **2.67** |  |  |
| Unspecified kidney failure | 10 | **2.30** | **1.09** | **4.24** |  |  |
| All kidney failure | 516 | **1.64** | **1.50** | **1.79** |  |  |
| Familial risks were adjusted for age, sex, time period, region of residence, socioeconomic status, and comorbidities. |  |  |  |  |  |  |
| Bold type: 95% CI does not include 1.00. O = observed number of cases with family history of kidney failure; SIR = standardized incidence ratio; CI = confidence interval | | | | | |  |
