# Supplementary material for: Familial Risks of Kidney Failure in Sweden: A Nationwide Family Study
Source: PLoS One. 2014 Nov 25;9(11):e113353. doi: 10.1371/journal.pone.0113353 (PMC4244139; doi:10.1371/journal.pone.0113353)
Supplement: Table S2 — Familial risk of concordant and discordant kidney failure in men and women. (DOCX) [file pone.0113353.s002.docx]

| **Table S2. Familial risk of concordant and discordant kidney failure in men and women** | | | | | | | | | | | | | | |  |
| --- | --- | --- | --- | --- | --- | --- | --- | --- | --- | --- | --- | --- | --- | --- | --- |
|  |  | Males | | | |  | Females | | | |  | All | | | |
| Probands with any type of kidney failure | Subtype of kidney failure in offspring/siblings | O | SIR | 95% CI | |  | O | SIR | 95% CI | |  | O | SIR | 95% CI | |
| **Family history (parent/sibling)** |  |  |  |  |  |  |  |  |  |  |  |  |  |  |  |
| Acute kidney failure | Acute kidney failure | 153 | 1.09 | 0.92 | 1.27 |  | 84 | 1.05 | 0.84 | 1.30 |  | 237 | 1.08 | 0.94 | 1.22 |
|  | Chronic kidney failure | 282 | 1.07 | 0.95 | 1.20 |  | 153 | 1.15 | 0.98 | 1.35 |  | 435 | **1.10** | **1.00** | **1.21** |
|  | Unspecified kidney failure | 64 | **1.29** | **1.00** | **1.65** |  | 36 | 1.31 | 0.92 | 1.81 |  | 100 | **1.30** | **1.06** | **1.58** |
|  | All kidney failure | 499 | **1.10** | **1.01** | **1.20** |  | 273 | **1.14** | **1.01** | **1.28** |  | 772 | **1.11** | **1.04** | **1.19** |
|  |  |  |  |  |  |  |  |  |  |  |  |  |  |  |  |
| Chronic kidney failure | Acute kidney failure | 201 | 1.15 | 0.99 | 1.32 |  | 129 | **1.26** | **1.05** | **1.50** |  | 330 | **1.19** | **1.06** | **1.32** |
|  | Chronic kidney failure | 717 | **2.04** | **1.90** | **2.20** |  | 395 | **1.97** | **1.78** | **2.17** |  | 1112 | **2.02** | **1.90** | **2.14** |
|  | Unspecified kidney failure | 104 | **1.56** | **1.28** | **1.89** |  | 65 | **1.76** | **1.36** | **2.24** |  | 169 | **1.63** | **1.40** | **1.90** |
|  | All kidney failure | 1022 | **1.72** | **1.62** | **1.83** |  | 589 | **1.73** | **1.59** | **1.88** |  | 1611 | **1.73** | **1.64** | **1.81** |
|  |  |  |  |  |  |  |  |  |  |  |  |  |  |  |  |
| Unspecified kidney failure | Acute kidney failure | 72 | 1.07 | 0.84 | 1.35 |  | 42 | 1.04 | 0.75 | 1.41 |  | 114 | 1.06 | 0.88 | 1.28 |
|  | Chronic kidney failure | 176 | **1.31** | **1.12** | **1.52** |  | 101 | **1.31** | **1.07** | **1.60** |  | 277 | **1.31** | **1.16** | **1.47** |
|  | Unspecified kidney failure | 33 | 1.18 | 0.81 | 1.65 |  | 21 | 1.38 | 0.85 | 2.11 |  | 54 | 1.25 | 0.94 | 1.63 |
|  | All kidney failure | 281 | **1.22** | **1.09** | **1.38** |  | 164 | **1.24** | **1.06** | **1.44** |  | 445 | **1.23** | **1.12** | **1.35** |
|  |  |  |  |  |  |  |  |  |  |  |  |  |  |  |  |
| All kidney failure | Acute kidney failure | 426 | **1.11** | **1.01** | **1.22** |  | 255 | **1.15** | **1.01** | **1.30** |  | 681 | **1.12** | **1.04** | **1.21** |
|  | Chronic kidney failure | 1175 | **1.57** | **1.48** | **1.66** |  | 649 | **1.58** | **1.46** | **1.71** |  | 1824 | **1.57** | **1.50** | **1.65** |
|  | Unspecified kidney failure | 201 | **1.39** | **1.21** | **1.60** |  | 122 | **1.53** | **1.27** | **1.83** |  | 323 | **1.44** | **1.29** | **1.61** |
|  | All kidney failure | 1802 | **1.41** | **1.35** | **1.48** |  | 1026 | **1.44** | **1.35** | **1.53** |  | 2828 | **1.42** | **1.37** | **1.48** |
| **Parents history** |  |  |  |  |  |  |  |  |  |  |  |  |  |  |  |
| Acute kidney failure | Acute kidney failure | 103 | 1.06 | 0.87 | 1.29 |  | 58 | 1.10 | 0.83 | 1.42 |  | 161 | 1.07 | 0.91 | 1.25 |
|  | Chronic kidney failure | 189 | 1.01 | 0.87 | 1.17 |  | 112 | 1.16 | 0.95 | 1.39 |  | 301 | 1.06 | 0.95 | 1.19 |
|  | Unspecified kidney failure | 49 | **1.37** | **1.01** | **1.81** |  | 24 | 1.23 | 0.79 | 1.83 |  | 73 | **1.32** | **1.03** | **1.66** |
|  | All kidney failure | 341 | 1.07 | 0.96 | 1.19 |  | 194 | 1.15 | 0.99 | 1.32 |  | 535 | **1.10** | **1.00** | **1.19** |
|  |  |  |  |  |  |  |  |  |  |  |  |  |  |  |  |
| Chronic kidney failure | Acute kidney failure | 118 | 1.07 | 0.89 | 1.28 |  | 84 | **1.40** | **1.12** | **1.73** |  | 202 | **1.19** | **1.03** | **1.36** |
|  | Chronic kidney failure | 378 | **1.71** | **1.54** | **1.89** |  | 204 | **1.62** | **1.40** | **1.85** |  | 582 | **1.67** | **1.54** | **1.82** |
|  | Unspecified kidney failure | 68 | **1.72** | **1.33** | **2.18** |  | 31 | 1.37 | 0.93 | 1.95 |  | 99 | **1.59** | **1.29** | **1.94** |
|  | All kidney failure | 564 | **1.52** | **1.40** | **1.65** |  | 319 | **1.53** | **1.36** | **1.71** |  | 883 | **1.52** | **1.42** | **1.63** |
|  |  |  |  |  |  |  |  |  |  |  |  |  |  |  |  |
| Unspecified kidney failure | Acute kidney failure | 59 | 1.09 | 0.83 | 1.40 |  | 32 | 1.01 | 0.69 | 1.43 |  | 91 | 1.06 | 0.85 | 1.30 |
|  | Chronic kidney failure | 138 | **1.23** | **1.03** | **1.45** |  | 76 | 1.21 | 0.96 | 1.52 |  | 214 | **1.22** | **1.06** | **1.40** |
|  | Unspecified kidney failure | 22 | 1.00 | 0.63 | 1.52 |  | 16 | 1.50 | 0.86 | 2.45 |  | 38 | 1.16 | 0.82 | 1.60 |
|  | All kidney failure | 219 | **1.16** | **1.01** | **1.32** |  | 124 | 1.18 | 0.98 | 1.41 |  | 343 | **1.17** | **1.05** | **1.30** |
|  |  |  |  |  |  |  |  |  |  |  |  |  |  |  |  |
| All kidney failure | Acute kidney failure | 280 | 1.07 | 0.95 | 1.20 |  | 174 | **1.20** | **1.03** | **1.40** |  | 454 | **1.12** | **1.02** | **1.23** |
|  | Chronic kidney failure | 705 | **1.36** | **1.26** | **1.46** |  | 392 | **1.37** | **1.24** | **1.52** |  | 1097 | **1.36** | **1.28** | **1.44** |
|  | Unspecified kidney failure | 139 | **1.43** | **1.20** | **1.69** |  | 71 | **1.35** | **1.05** | **1.70** |  | 210 | **1.40** | **1.22** | **1.60** |
|  | All kidney failure | 1124 | **1.28** | **1.21** | **1.36** |  | 637 | **1.32** | **1.22** | **1.43** |  | 1761 | **1.29** | **1.23** | **1.35** |
| **Paternal history** |  |  |  |  |  |  |  |  |  |  |  |  |  |  |  |
| Acute kidney failure | Acute kidney failure | 62 | 1.20 | 0.92 | 1.54 |  | 26 | 1.03 | 0.67 | 1.51 |  | 88 | 1.14 | 0.92 | 1.41 |
|  | Chronic kidney failure | 96 | 1.03 | 0.83 | 1.26 |  | 53 | 1.01 | 0.76 | 1.32 |  | 149 | 1.02 | 0.86 | 1.20 |
|  | Unspecified kidney failure | 27 | 1.49 | 0.98 | 2.18 |  | 15 | 1.31 | 0.73 | 2.17 |  | 42 | **1.42** | **1.03** | **1.93** |
|  | All kidney failure | 185 | 1.13 | 0.98 | 1.31 |  | 94 | 1.06 | 0.85 | 1.29 |  | 279 | 1.11 | 0.98 | 1.24 |
|  |  |  |  |  |  |  |  |  |  |  |  |  |  |  |  |
| Chronic kidney failure | Acute kidney failure | 82 | **1.32** | **1.05** | **1.64** |  | 43 | 1.21 | 0.87 | 1.63 |  | 125 | **1.28** | **1.07** | **1.53** |
|  | Chronic kidney failure | 212 | **1.73** | **1.50** | **1.98** |  | 105 | **1.45** | **1.19** | **1.76** |  | 317 | **1.62** | **1.45** | **1.81** |
|  | Unspecified kidney failure | 41 | **1.91** | **1.37** | **2.59** |  | 18 | 1.40 | 0.83 | 2.22 |  | 59 | **1.72** | **1.31** | **2.22** |
|  | All kidney failure | 335 | **1.63** | **1.46** | **1.81** |  | 166 | **1.37** | **1.17** | **1.60** |  | 501 | **1.53** | **1.40** | **1.67** |
|  |  |  |  |  |  |  |  |  |  |  |  |  |  |  |  |
| Unspecified kidney failure | Acute kidney failure | 30 | 1.00 | 0.67 | 1.43 |  | 19 | 1.13 | 0.68 | 1.77 |  | 49 | 1.05 | 0.77 | 1.38 |
|  | Chronic kidney failure | 78 | 1.26 | 0.99 | 1.57 |  | 36 | 0.96 | 0.67 | 1.33 |  | 114 | 1.14 | 0.94 | 1.38 |
|  | Unspecified kidney failure | 9 | 0.74 | 0.33 | 1.40 |  | 11 | 1.87 | 0.93 | 3.36 |  | 20 | 1.10 | 0.67 | 1.71 |
|  | All kidney failure | 117 | 1.12 | 0.93 | 1.34 |  | 66 | 1.09 | 0.85 | 1.39 |  | 183 | 1.11 | 0.96 | 1.29 |
|  |  |  |  |  |  |  |  |  |  |  |  |  |  |  |  |
| All kidney failure | Acute kidney failure | 174 | **1.21** | **1.04** | **1.41** |  | 88 | 1.13 | 0.91 | 1.40 |  | 262 | **1.18** | **1.04** | **1.34** |
|  | Chronic kidney failure | 386 | **1.39** | **1.25** | **1.53** |  | 194 | **1.19** | **1.03** | **1.38** |  | 580 | **1.32** | **1.21** | **1.43** |
|  | Unspecified kidney failure | 77 | **1.49** | **1.17** | **1.86** |  | 44 | **1.46** | **1.06** | **1.96** |  | 121 | **1.48** | **1.23** | **1.77** |
|  | All kidney failure | 637 | **1.35** | **1.24** | **1.45** |  | 326 | **1.21** | **1.08** | **1.35** |  | 963 | **1.29** | **1.21** | **1.38** |
| **Maternal history** |  |  |  |  |  |  |  |  |  |  |  |  |  |  |  |
| Acute kidney failure | Acute kidney failure | 42 | 0.92 | 0.66 | 1.25 |  | 33 | 1.19 | 0.82 | 1.67 |  | 75 | 1.02 | 0.80 | 1.28 |
|  | Chronic kidney failure | 94 | 0.97 | 0.79 | 1.19 |  | 59 | 1.29 | 0.98 | 1.66 |  | 153 | 1.07 | 0.91 | 1.26 |
|  | Unspecified kidney failure | 23 | 1.26 | 0.80 | 1.89 |  | 9 | 1.05 | 0.48 | 2.00 |  | 32 | 1.19 | 0.81 | 1.68 |
|  | All kidney failure | 159 | 0.99 | 0.84 | 1.16 |  | 101 | **1.23** | **1.00** | **1.49** |  | 260 | 1.07 | 0.95 | 1.21 |
|  |  |  |  |  |  |  |  |  |  |  |  |  |  |  |  |
| Chronic kidney failure | Acute kidney failure | 38 | 0.70 | 0.50 | 0.97 |  | 42 | **1.64** | **1.18** | **2.22** |  | 80 | 1.01 | 0.80 | 1.25 |
|  | Chronic kidney failure | 178 | **1.69** | **1.45** | **1.96** |  | 108 | **1.88** | **1.54** | **2.27** |  | 286 | **1.76** | **1.56** | **1.97** |
|  | Unspecified kidney failure | 29 | **1.55** | **1.04** | **2.23** |  | 13 | 1.24 | 0.66 | 2.12 |  | 42 | **1.44** | **1.04** | **1.95** |
|  | All kidney failure | 245 | **1.38** | **1.21** | **1.56** |  | 163 | **1.74** | **1.49** | **2.03** |  | 408 | **1.50** | **1.36** | **1.66** |
|  |  |  |  |  |  |  |  |  |  |  |  |  |  |  |  |
| Unspecified kidney failure | Acute kidney failure | 30 | 1.18 | 0.80 | 1.69 |  | 13 | 0.89 | 0.47 | 1.52 |  | 43 | 1.08 | 0.78 | 1.45 |
|  | Chronic kidney failure | 66 | 1.23 | 0.95 | 1.56 |  | 42 | **1.56** | **1.12** | **2.11** |  | 108 | **1.34** | **1.10** | **1.62** |
|  | Unspecified kidney failure | 13 | 1.26 | 0.67 | 2.16 |  | 7 | 1.39 | 0.55 | 2.89 |  | 20 | 1.31 | 0.80 | 2.02 |
|  | All kidney failure | 109 | **1.22** | **1.00** | **1.47** |  | 62 | **1.33** | **1.02** | **1.71** |  | 171 | **1.26** | **1.08** | **1.46** |
|  |  |  |  |  |  |  |  |  |  |  |  |  |  |  |  |
| All kidney failure | Acute kidney failure | 110 | 0.88 | 0.72 | 1.06 |  | 88 | **1.29** | **1.04** | **1.59** |  | 198 | 1.03 | 0.89 | 1.18 |
|  | Chronic kidney failure | 338 | **1.32** | **1.19** | **1.47** |  | 209 | **1.60** | **1.39** | **1.84** |  | 547 | **1.42** | **1.30** | **1.54** |
|  | Unspecified kidney failure | 65 | **1.38** | **1.06** | **1.75** |  | 29 | 1.20 | 0.80 | 1.73 |  | 94 | **1.32** | **1.06** | **1.61** |
|  | All kidney failure | 513 | **1.20** | **1.10** | **1.31** |  | 326 | **1.47** | **1.31** | **1.63** |  | 839 | **1.29** | **1.21** | **1.38** |
| **Sibling history** |  |  |  |  |  |  |  |  |  |  |  |  |  |  |  |
| Acute kidney failure | Acute kidney failure | 52 | 1.15 | 0.86 | 1.50 |  | 27 | 0.98 | 0.64 | 1.43 |  | 79 | 1.08 | 0.86 | 1.35 |
|  | Chronic kidney failure | 97 | 1.19 | 0.97 | 1.45 |  | 42 | 1.09 | 0.79 | 1.47 |  | 139 | 1.16 | 0.97 | 1.37 |
|  | Unspecified kidney failure | 16 | 1.08 | 0.62 | 1.77 |  | 12 | 1.44 | 0.74 | 2.52 |  | 28 | 1.21 | 0.80 | 1.75 |
|  | All kidney failure | 165 | 1.17 | 0.99 | 1.36 |  | 81 | 1.09 | 0.86 | 1.35 |  | 246 | **1.14** | **1.00** | **1.29** |
|  |  |  |  |  |  |  |  |  |  |  |  |  |  |  |  |
| Chronic kidney failure | Acute kidney failure | 88 | **1.25** | **1.00** | **1.54** |  | 49 | 1.09 | 0.81 | 1.44 |  | 137 | **1.19** | **1.00** | **1.40** |
|  | Chronic kidney failure | 366 | **2.52** | **2.27** | **2.80** |  | 213 | **2.52** | **2.19** | **2.88** |  | 579 | **2.52** | **2.32** | **2.73** |
|  | Unspecified kidney failure | 42 | **1.42** | **1.02** | **1.92** |  | 36 | **2.14** | **1.50** | **2.97** |  | 78 | **1.68** | **1.33** | **2.10** |
|  | All kidney failure | 496 | **2.02** | **1.85** | **2.21** |  | 298 | **2.04** | **1.81** | **2.28** |  | 794 | **2.03** | **1.89** | **2.18** |
|  |  |  |  |  |  |  |  |  |  |  |  |  |  |  |  |
| Unspecified kidney failure | Acute kidney failure | 17 | 1.10 | 0.64 | 1.77 |  | 13 | 1.32 | 0.70 | 2.26 |  | 30 | 1.19 | 0.80 | 1.70 |
|  | Chronic kidney failure | 48 | **1.58** | **1.16** | **2.09** |  | 32 | **1.86** | **1.27** | **2.63** |  | 80 | **1.68** | **1.33** | **2.09** |
|  | Unspecified kidney failure | 12 | 1.65 | 0.85 | 2.89 |  | 6 | 1.08 | 0.39 | 2.37 |  | 18 | 1.40 | 0.83 | 2.22 |
|  | All kidney failure | 77 | **1.45** | **1.14** | **1.81** |  | 51 | **1.56** | **1.16** | **2.06** |  | 128 | **1.49** | **1.25** | **1.78** |
|  |  |  |  |  |  |  |  |  |  |  |  |  |  |  |  |
| All kidney failure | Acute kidney failure | 157 | **1.20** | **1.02** | **1.40** |  | 89 | 1.08 | 0.87 | 1.33 |  | 246 | **1.15** | **1.01** | **1.31** |
|  | Chronic kidney failure | 511 | **1.99** | **1.82** | **2.17** |  | 287 | **2.04** | **1.81** | **2.30** |  | 798 | **2.01** | **1.87** | **2.15** |
|  | Unspecified kidney failure | 70 | **1.36** | **1.06** | **1.72** |  | 54 | **1.76** | **1.32** | **2.30** |  | 124 | **1.51** | **1.25** | **1.80** |
|  | All kidney failure | 738 | **1.68** | **1.56** | **1.80** |  | 430 | **1.70** | **1.54** | **1.87** |  | 1168 | **1.69** | **1.59** | **1.78** |
| Familial risks were adjusted for age, sex, time period, region of residence, socioeconomic status, and comorbidities.  Bold type: 95% CI does not include 1.00. | |  |  |  |  |  |  |  |  |  |  |  |  |  |  |
| O = observed number of cases with family history of kidney failure; SIR = standardized incidence ratio; CI = confidence interval | | | | |  |  |  |  |  |  |  |  |  |  |  |
