# Supplementary material for: Familial Risks of Kidney Failure in Sweden: A Nationwide Family Study
Source: PLoS One. 2014 Nov 25;9(11):e113353. doi: 10.1371/journal.pone.0113353 (PMC4244139; doi:10.1371/journal.pone.0113353)
Supplement: Table S3 — Familial risk (sibling/parent history) of concordant and discordant kidney failure in males and females by age at diagnosis. (DOCX) [file pone.0113353.s003.docx]

| **Table S3. Familial risk (sibling/parent history) of concordant and discordant kidney failure in males and females by age at diagnosis** | | | | | | | | | | | | | | | |
| --- | --- | --- | --- | --- | --- | --- | --- | --- | --- | --- | --- | --- | --- | --- | --- |
|  |  | Males | | | |  | Females | | | |  | All | | | |
| Kidney failure in probands | Age at diagnosis (years) of  subtype of kidney failure | O | SIR | 95% CI | |  | O | SIR | 95% CI | |  | O | SIR | 95% CI | |
| Acute kidney failure | Acute kidney failure |  |  |  |  |  |  |  |  |  |  |  |  |  |  |
|  | <10 | 4 | **16.78** | **4.36** | **43.39** |  | 2 | **10.88** | **1.03** | **40.02** |  | 6 | **14.21** | **5.11** | **31.14** |
|  | 10-19 | 0 |  |  |  |  | 0 |  |  |  |  | 0 |  |  |  |
|  | 20-29 | 6 | 2.00 | 0.72 | 4.37 |  | 5 | **3.70** | **1.17** | **8.70** |  | 11 | **2.52** | **1.25** | **4.53** |
|  | 30-39 | 11 | 1.43 | 0.71 | 2.57 |  | 2 | 0.61 | 0.06 | 2.24 |  | 13 | 1.18 | 0.63 | 2.03 |
|  | 40-49 | 22 | 1.23 | 0.77 | 1.87 |  | 14 | 1.57 | 0.86 | 2.64 |  | 36 | 1.34 | 0.94 | 1.86 |
|  | 50-59 | 52 | **1.35** | **1.01** | **1.78** |  | 21 | 0.84 | 0.52 | 1.28 |  | 73 | 1.15 | 0.90 | 1.45 |
|  | >=60 | 58 | 0.80 | 0.61 | 1.03 |  | 40 | 0.99 | 0.71 | 1.35 |  | 98 | 0.87 | 0.70 | 1.06 |
|  | All | 153 | 1.09 | 0.92 | 1.27 |  | 84 | 1.05 | 0.84 | 1.30 |  | 237 | 1.08 | 0.94 | 1.22 |
|  | Chronic kidney failure |  |  |  |  |  |  |  |  |  |  |  |  |  |  |
|  | <10 | 0 |  |  |  |  | 1 | 5.59 | 0.00 | 32.04 |  | 1 | 2.23 | 0.00 | 12.77 |
|  | 10-19 | 1 | 0.99 | 0.00 | 5.67 |  | 2 | 3.63 | 0.34 | 13.36 |  | 3 | 1.92 | 0.36 | 5.69 |
|  | 20-29 | 3 | 0.59 | 0.11 | 1.75 |  | 2 | 0.54 | 0.05 | 2.00 |  | 5 | 0.57 | 0.18 | 1.34 |
|  | 30-39 | 21 | 1.10 | 0.68 | 1.68 |  | 15 | 1.25 | 0.70 | 2.07 |  | 36 | 1.16 | 0.81 | 1.60 |
|  | 40-49 | 43 | 0.98 | 0.71 | 1.32 |  | 26 | 1.09 | 0.71 | 1.60 |  | 69 | 1.02 | 0.79 | 1.29 |
|  | 50-59 | 95 | 1.19 | 0.96 | 1.45 |  | 53 | **1.40** | **1.05** | **1.83** |  | 148 | **1.26** | **1.06** | **1.48** |
|  | >=60 | 119 | 1.04 | 0.86 | 1.25 |  | 54 | 0.99 | 0.74 | 1.29 |  | 173 | 1.02 | 0.88 | 1.19 |
|  | All | 282 | 1.07 | 0.95 | 1.20 |  | 153 | 1.15 | 0.98 | 1.35 |  | 435 | **1.10** | **1.00** | **1.21** |
|  | Unspecified kidney failure |  |  |  |  |  |  |  |  |  |  |  |  |  |  |
|  | <10 | 0 |  |  |  |  | 0 |  |  |  |  | 0 |  |  |  |
|  | 10-19 | 0 |  |  |  |  | 0 |  |  |  |  | 0 |  |  |  |
|  | 20-29 | 2 | 3.07 | 0.29 | 11.31 |  | 0 |  |  |  |  | 2 | 1.74 | 0.16 | 6.41 |
|  | 30-39 | 2 | 0.76 | 0.07 | 2.80 |  | 0 |  |  |  |  | 2 | 0.44 | 0.04 | 1.61 |
|  | 40-49 | 7 | 0.90 | 0.36 | 1.86 |  | 7 | 1.15 | 0.46 | 2.39 |  | 14 | 1.01 | 0.55 | 1.70 |
|  | 50-59 | 19 | 1.35 | 0.81 | 2.11 |  | 9 | 1.16 | 0.53 | 2.22 |  | 28 | 1.28 | 0.85 | 1.86 |
|  | >=60 | 34 | 1.41 | 0.97 | 1.97 |  | 20 | **1.80** | **1.10** | **2.78** |  | 54 | **1.53** | **1.15** | **2.00** |
|  | All | 64 | **1.29** | **1.00** | **1.65** |  | 36 | 1.31 | 0.92 | 1.81 |  | 100 | **1.30** | **1.06** | **1.58** |
|  | All kidney failure |  |  |  |  |  |  |  |  |  |  |  |  |  |  |
|  | <10 | 4 | **7.27** | **1.89** | **18.79** |  | 3 | **7.25** | **1.37** | **21.47** |  | 7 | **7.26** | **2.88** | **15.04** |
|  | 10-19 | 1 | 0.51 | 0.00 | 2.90 |  | 2 | 1.52 | 0.14 | 5.60 |  | 3 | 0.91 | 0.17 | 2.70 |
|  | 20-29 | 11 | 1.26 | 0.62 | 2.26 |  | 7 | 1.26 | 0.50 | 2.62 |  | 18 | 1.26 | 0.75 | 2.00 |
|  | 30-39 | 34 | 1.15 | 0.80 | 1.61 |  | 17 | 0.99 | 0.57 | 1.58 |  | 51 | 1.09 | 0.81 | 1.44 |
|  | 40-49 | 72 | 1.04 | 0.81 | 1.31 |  | 47 | 1.21 | 0.89 | 1.61 |  | 119 | 1.10 | 0.91 | 1.32 |
|  | 50-59 | 166 | **1.25** | **1.07** | **1.46** |  | 83 | 1.17 | 0.94 | 1.46 |  | 249 | **1.23** | **1.08** | **1.39** |
|  | >=60 | 211 | 1.00 | 0.87 | 1.14 |  | 114 | 1.07 | 0.89 | 1.29 |  | 325 | 1.02 | 0.92 | 1.14 |
|  | All | 499 | **1.10** | **1.01** | **1.20** |  | 273 | **1.14** | **1.01** | **1.28** |  | 772 | **1.11** | **1.04** | **1.19** |
| Chronic kidney failure | Acute kidney failure |  |  |  |  |  |  |  |  |  |  |  |  |  |  |
|  | <10 | 0 |  |  |  |  | 3 | **6.15** | **1.16** | **18.22** |  | 3 | 3.06 | 0.58 | 9.07 |
|  | 10-19 | 0 |  |  |  |  | 3 | 1.61 | 0.30 | 4.77 |  | 3 | 0.93 | 0.18 | 2.76 |
|  | 20-29 | 8 | 1.36 | 0.58 | 2.69 |  | 3 | 0.99 | 0.19 | 2.93 |  | 11 | 1.23 | 0.61 | 2.22 |
|  | 30-39 | 19 | 1.18 | 0.71 | 1.85 |  | 7 | 1.18 | 0.47 | 2.45 |  | 26 | 1.18 | 0.77 | 1.74 |
|  | 40-49 | 34 | 1.44 | 0.99 | 2.01 |  | 17 | 1.18 | 0.68 | 1.89 |  | 51 | **1.34** | **1.00** | **1.76** |
|  | 50-59 | 59 | 1.23 | 0.94 | 1.59 |  | 29 | 1.03 | 0.69 | 1.48 |  | 88 | 1.16 | 0.93 | 1.42 |
|  | >=60 | 81 | 1.02 | 0.81 | 1.26 |  | 67 | **1.38** | **1.07** | **1.75** |  | 148 | 1.15 | 0.97 | 1.35 |
|  | All | 201 | 1.15 | 0.99 | 1.32 |  | 129 | **1.26** | **1.05** | **1.50** |  | 330 | **1.19** | **1.06** | **1.32** |
|  | Chronic kidney failure |  |  |  |  |  |  |  |  |  |  |  |  |  |  |
|  | <10 | 2 | 3.92 | 0.37 | 14.41 |  | 0 |  |  |  |  | 2 | 2.09 | 0.20 | 7.70 |
|  | 10-19 | 16 | **6.94** | **3.96** | **11.30** |  | 11 | **5.60** | **2.78** | **10.06** |  | 27 | **6.33** | **4.16** | **9.22** |
|  | 20-29 | 41 | **4.87** | **3.49** | **6.61** |  | 33 | **4.35** | **2.99** | **6.12** |  | 74 | **4.62** | **3.63** | **5.81** |
|  | 30-39 | 91 | **2.36** | **1.90** | **2.90** |  | 43 | **1.76** | **1.27** | **2.37** |  | 134 | **2.13** | **1.78** | **2.52** |
|  | 40-49 | 135 | **2.08** | **1.75** | **2.47** |  | 75 | **2.11** | **1.66** | **2.65** |  | 210 | **2.09** | **1.82** | **2.40** |
|  | 50-59 | 187 | **1.86** | **1.60** | **2.14** |  | 92 | **1.74** | **1.40** | **2.14** |  | 279 | **1.82** | **1.61** | **2.04** |
|  | >=60 | 245 | **1.81** | **1.59** | **2.05** |  | 141 | **1.81** | **1.52** | **2.14** |  | 386 | **1.81** | **1.63** | **2.00** |
|  | All | 717 | **2.04** | **1.90** | **2.20** |  | 395 | **1.97** | **1.78** | **2.17** |  | 1112 | **2.02** | **1.90** | **2.14** |
|  | Unspecified kidney failure |  |  |  |  |  |  |  |  |  |  |  |  |  |  |
|  | <10 | 0 |  |  |  |  | 0 |  |  |  |  | 0 |  |  |  |
|  | 10-19 | 4 | **13.55** | **3.52** | **35.03** |  | 2 | 9.12 | 0.86 | 33.52 |  | 6 | **11.66** | **4.20** | **25.54** |
|  | 20-29 | 4 | 2.44 | 0.63 | 6.30 |  | 5 | **4.40** | **1.39** | **10.35** |  | 9 | **3.24** | **1.47** | **6.18** |
|  | 30-39 | 15 | **3.12** | **1.74** | **5.17** |  | 9 | **2.56** | **1.16** | **4.88** |  | 24 | **2.88** | **1.85** | **4.30** |
|  | 40-49 | 18 | **1.76** | **1.04** | **2.79** |  | 14 | **2.48** | **1.35** | **4.17** |  | 32 | **2.01** | **1.38** | **2.85** |
|  | 50-59 | 27 | 1.38 | 0.91 | 2.01 |  | 15 | 1.39 | 0.77 | 2.30 |  | 42 | **1.38** | **1.00** | **1.87** |
|  | >=60 | 36 | 1.20 | 0.84 | 1.67 |  | 20 | 1.29 | 0.79 | 2.00 |  | 56 | 1.23 | 0.93 | 1.60 |
|  | All | 104 | **1.56** | **1.28** | **1.89** |  | 65 | **1.76** | **1.36** | **2.24** |  | 169 | **1.63** | **1.40** | **1.90** |
|  | All kidney failure |  |  |  |  |  |  |  |  |  |  |  |  |  |  |
|  | <10 | 2 | 1.82 | 0.17 | 6.70 |  | 3 | 2.73 | 0.51 | 8.08 |  | 5 | 2.28 | 0.72 | 5.36 |
|  | 10-19 | 20 | **5.05** | **3.08** | **7.81** |  | 16 | **3.96** | **2.26** | **6.44** |  | 36 | **4.50** | **3.15** | **6.23** |
|  | 20-29 | 53 | **3.32** | **2.49** | **4.35** |  | 41 | **3.49** | **2.50** | **4.74** |  | 94 | **3.39** | **2.74** | **4.15** |
|  | 30-39 | 125 | **2.11** | **1.75** | **2.51** |  | 59 | **1.74** | **1.32** | **2.25** |  | 184 | **1.97** | **1.70** | **2.28** |
|  | 40-49 | 187 | **1.89** | **1.63** | **2.19** |  | 106 | **1.91** | **1.56** | **2.31** |  | 293 | **1.90** | **1.69** | **2.13** |
|  | 50-59 | 273 | **1.62** | **1.43** | **1.83** |  | 136 | **1.48** | **1.24** | **1.75** |  | 409 | **1.57** | **1.42** | **1.73** |
|  | >=60 | 362 | **1.48** | **1.33** | **1.64** |  | 228 | **1.61** | **1.41** | **1.83** |  | 590 | **1.52** | **1.40** | **1.65** |
|  | All | 1022 | **1.72** | **1.62** | **1.83** |  | 589 | **1.73** | **1.59** | **1.88** |  | 1611 | **1.73** | **1.64** | **1.81** |
| Unspecified kidney failure | Acute kidney failure |  |  |  |  |  |  |  |  |  |  |  |  |  |  |
|  | <10 | 0 |  |  |  |  | 0 |  |  |  |  | 0 |  |  |  |
|  | 10-19 | 0 |  |  |  |  | 2 | 7.22 | 0.68 | 26.56 |  | 2 | 4.33 | 0.41 | 15.91 |
|  | 20-29 | 0 |  |  |  |  | 2 | 3.67 | 0.35 | 13.49 |  | 2 | 1.17 | 0.11 | 4.31 |
|  | 30-39 | 2 | 0.72 | 0.07 | 2.65 |  | 0 | 0.00 | 0.61 | 2.47 |  | 2 | 0.46 | 0.04 | 1.69 |
|  | 40-49 | 8 | 1.02 | 0.44 | 2.03 |  | 3 | 0.83 | 0.16 | 2.45 |  | 11 | 0.96 | 0.48 | 1.73 |
|  | 50-59 | 22 | 1.15 | 0.72 | 1.74 |  | 9 | 0.74 | 0.33 | 1.41 |  | 31 | 0.99 | 0.67 | 1.40 |
|  | >=60 | 40 | 1.12 | 0.80 | 1.52 |  | 26 | 1.18 | 0.77 | 1.73 |  | 66 | 1.14 | 0.88 | 1.45 |
|  | All | 72 | 1.07 | 0.84 | 1.35 |  | 42 | 1.04 | 0.75 | 1.41 |  | 114 | 1.06 | 0.88 | 1.28 |
|  | Chronic kidney failure |  |  |  |  |  |  |  |  |  |  |  |  |  |  |
|  | <10 | 0 |  |  |  |  | 0 |  |  |  |  | 0 |  |  |  |
|  | 10-19 | 2 | 4.55 | 0.43 | 16.72 |  | 4 | **13.32** | **3.46** | **34.43** |  | 6 | **8.10** | **2.92** | **17.76** |
|  | 20-29 | 7 | **3.16** | **1.25** | **6.55** |  | 5 | 2.90 | 0.91 | 6.81 |  | 12 | **3.04** | **1.57** | **5.34** |
|  | 30-39 | 13 | 1.31 | 0.69 | 2.24 |  | 10 | 1.84 | 0.88 | 3.40 |  | 23 | 1.49 | 0.95 | 2.25 |
|  | 40-49 | 21 | 1.06 | 0.65 | 1.62 |  | 18 | 1.47 | 0.87 | 2.32 |  | 39 | 1.21 | 0.86 | 1.66 |
|  | 50-59 | 46 | 1.25 | 0.92 | 1.67 |  | 28 | 1.31 | 0.87 | 1.89 |  | 74 | **1.27** | **1.00** | **1.60** |
|  | >=60 | 87 | **1.33** | **1.07** | **1.64** |  | 36 | 1.01 | 0.71 | 1.40 |  | 123 | **1.22** | **1.01** | **1.45** |
|  | All | 176 | **1.31** | **1.12** | **1.52** |  | 101 | **1.31** | **1.07** | **1.60** |  | 277 | **1.31** | **1.16** | **1.47** |
|  | Unspecified kidney failure |  |  |  |  |  |  |  |  |  |  |  |  |  |  |
|  | <10 | 0 |  |  |  |  | 0 |  |  |  |  | 0 |  |  |  |
|  | 10-19 | 0 |  |  |  |  | 0 |  |  |  |  | 0 |  |  |  |
|  | 20-29 | 1 | 2.39 | 0.00 | 13.67 |  | 1 | 4.41 | 0.00 | 25.26 |  | 2 | 3.10 | 0.29 | 11.38 |
|  | 30-39 | 6 | **2.80** | **1.01** | **6.12** |  | 1 | 1.04 | 0.00 | 5.95 |  | 7 | 2.25 | 0.89 | 4.66 |
|  | 40-49 | 2 | 0.58 | 0.05 | 2.12 |  | 4 | 1.62 | 0.42 | 4.18 |  | 6 | 1.01 | 0.36 | 2.21 |
|  | 50-59 | 8 | 1.04 | 0.45 | 2.07 |  | 4 | 1.18 | 0.31 | 3.05 |  | 12 | 1.09 | 0.56 | 1.90 |
|  | >=60 | 16 | 1.13 | 0.64 | 1.83 |  | 11 | 1.35 | 0.67 | 2.43 |  | 27 | 1.21 | 0.80 | 1.76 |
|  | All | 33 | 1.18 | 0.81 | 1.65 |  | 21 | 1.38 | 0.85 | 2.11 |  | 54 | 1.25 | 0.94 | 1.63 |
|  | All kidney failure |  |  |  |  |  |  |  |  |  |  |  |  |  |  |
|  | <10 | 0 |  |  |  |  | 0 |  |  |  |  | 0 |  |  |  |
|  | 10-19 | 2 | 2.59 | 0.24 | 9.53 |  | 6 | **9.67** | **3.48** | **21.19** |  | 8 | **5.75** | **2.45** | **11.38** |
|  | 20-29 | 8 | 2.11 | 0.90 | 4.17 |  | 8 | **3.20** | **1.37** | **6.34** |  | 16 | **2.54** | **1.45** | **4.14** |
|  | 30-39 | 21 | 1.41 | 0.87 | 2.16 |  | 11 | 1.38 | 0.68 | 2.47 |  | 32 | 1.40 | 0.96 | 1.98 |
|  | 40-49 | 31 | 1.00 | 0.68 | 1.42 |  | 25 | 1.36 | 0.88 | 2.01 |  | 56 | 1.13 | 0.85 | 1.47 |
|  | 50-59 | 76 | 1.20 | 0.94 | 1.50 |  | 41 | 1.11 | 0.79 | 1.50 |  | 117 | 1.16 | 0.96 | 1.40 |
|  | >=60 | 143 | **1.24** | **1.05** | **1.46** |  | 73 | 1.11 | 0.87 | 1.39 |  | 216 | **1.19** | **1.04** | **1.36** |
|  | All | 281 | **1.22** | **1.09** | **1.38** |  | 164 | **1.24** | **1.06** | **1.44** |  | 445 | **1.23** | **1.12** | **1.35** |
| All kidney failure | Acute kidney failure |  |  |  |  |  |  |  |  |  |  |  |  |  |  |
|  | <10 | 4 | **5.00** | **1.30** | **12.93** |  | 5 | **6.76** | **2.13** | **15.90** |  | 9 | **5.84** | **2.65** | **11.14** |
|  | 10-19 | 0 |  |  |  |  | 5 | 1.78 | 0.56 | 4.20 |  | 5 | 0.97 | 0.31 | 2.28 |
|  | 20-29 | 14 | 1.39 | 0.76 | 2.34 |  | 10 | 2.03 | 0.97 | 3.74 |  | 24 | **1.60** | **1.03** | **2.39** |
|  | 30-39 | 32 | 1.21 | 0.83 | 1.71 |  | 9 | 0.83 | 0.38 | 1.59 |  | 41 | 1.10 | 0.79 | 1.49 |
|  | 40-49 | 64 | **1.30** | **1.00** | **1.66** |  | 34 | 1.26 | 0.87 | 1.76 |  | 98 | **1.28** | **1.04** | **1.57** |
|  | 50-59 | 133 | **1.26** | **1.05** | **1.49** |  | 59 | 0.90 | 0.69 | 1.17 |  | 192 | 1.12 | 0.97 | 1.29 |
|  | >=60 | 179 | 0.95 | 0.82 | 1.10 |  | 133 | **1.20** | **1.00** | **1.42** |  | 312 | 1.04 | 0.93 | 1.16 |
|  | All | 426 | **1.11** | **1.01** | **1.22** |  | 255 | **1.15** | **1.01** | **1.30** |  | 681 | **1.12** | **1.04** | **1.21** |
|  | Chronic kidney failure |  |  |  |  |  |  |  |  |  |  |  |  |  |  |
|  | <10 | 2 | 2.30 | 0.22 | 8.47 |  | 1 | 1.38 | 0.00 | 7.90 |  | 3 | 1.88 | 0.35 | 5.57 |
|  | 10-19 | 19 | **5.06** | **3.04** | **7.91** |  | 17 | **6.04** | **3.51** | **9.69** |  | 36 | **5.48** | **3.84** | **7.59** |
|  | 20-29 | 51 | **3.24** | **2.41** | **4.27** |  | 40 | **3.08** | **2.20** | **4.19** |  | 91 | **3.17** | **2.55** | **3.89** |
|  | 30-39 | 125 | **1.85** | **1.54** | **2.20** |  | 68 | **1.62** | **1.26** | **2.06** |  | 193 | **1.76** | **1.52** | **2.03** |
|  | 40-49 | 199 | **1.55** | **1.34** | **1.78** |  | 119 | **1.66** | **1.38** | **1.99** |  | 318 | **1.59** | **1.42** | **1.77** |
|  | 50-59 | 328 | **1.51** | **1.35** | **1.68** |  | 173 | **1.54** | **1.32** | **1.79** |  | 501 | **1.52** | **1.39** | **1.66** |
|  | >=60 | 451 | **1.43** | **1.30** | **1.57** |  | 231 | **1.37** | **1.20** | **1.56** |  | 682 | **1.41** | **1.31** | **1.52** |
|  | All | 1175 | **1.57** | **1.48** | **1.66** |  | 649 | **1.58** | **1.46** | **1.71** |  | 1824 | **1.57** | **1.50** | **1.65** |
|  | Unspecified kidney failure |  |  |  |  |  |  |  |  |  |  |  |  |  |  |
|  | <10 | 0 |  |  |  |  | 0 |  |  |  |  | 0 |  |  |  |
|  | 10-19 | 4 | **6.60** | **1.72** | **17.07** |  | 2 | 5.57 | 0.53 | 20.49 |  | 6 | **6.22** | **2.24** | **13.63** |
|  | 20-29 | 7 | **2.58** | **1.02** | **5.35** |  | 6 | **3.22** | **1.16** | **7.07** |  | 13 | **2.84** | **1.51** | **4.88** |
|  | 30-39 | 23 | **2.40** | **1.52** | **3.61** |  | 10 | 1.56 | 0.74 | 2.88 |  | 33 | **2.06** | **1.42** | **2.90** |
|  | 40-49 | 27 | 1.26 | 0.83 | 1.83 |  | 25 | **1.76** | **1.14** | **2.60** |  | 52 | **1.46** | **1.09** | **1.91** |
|  | 50-59 | 54 | 1.31 | 0.98 | 1.71 |  | 28 | 1.28 | 0.85 | 1.85 |  | 82 | **1.30** | **1.03** | **1.61** |
|  | >=60 | 86 | **1.26** | **1.01** | **1.56** |  | 51 | **1.47** | **1.09** | **1.93** |  | 137 | **1.33** | **1.12** | **1.57** |
|  | All | 201 | **1.39** | **1.21** | **1.60** |  | 122 | **1.53** | **1.27** | **1.83** |  | 323 | **1.44** | **1.29** | **1.61** |
|  | All kidney failure |  |  |  |  |  |  |  |  |  |  |  |  |  |  |
|  | <10 | 6 | **3.28** | **1.18** | **7.19** |  | 6 | **3.52** | **1.27** | **7.71** |  | 12 | **3.40** | **1.75** | **5.95** |
|  | 10-19 | 23 | **3.43** | **2.17** | **5.15** |  | 24 | **4.02** | **2.57** | **5.98** |  | 47 | **3.70** | **2.72** | **4.93** |
|  | 20-29 | 72 | **2.53** | **1.98** | **3.18** |  | 56 | **2.83** | **2.14** | **3.68** |  | 128 | **2.65** | **2.21** | **3.15** |
|  | 30-39 | 180 | **1.74** | **1.49** | **2.01** |  | 87 | **1.47** | **1.18** | **1.82** |  | 267 | **1.64** | **1.45** | **1.85** |
|  | 40-49 | 290 | **1.45** | **1.29** | **1.63** |  | 178 | **1.58** | **1.36** | **1.83** |  | 468 | **1.50** | **1.37** | **1.64** |
|  | 50-59 | 515 | **1.41** | **1.29** | **1.54** |  | 260 | **1.30** | **1.15** | **1.47** |  | 775 | **1.37** | **1.28** | **1.47** |
|  | >=60 | 716 | **1.25** | **1.16** | **1.35** |  | 415 | **1.32** | **1.20** | **1.46** |  | 1131 | **1.28** | **1.20** | **1.35** |
|  | All | 1802 | **1.41** | **1.35** | **1.48** |  | 1026 | **1.44** | **1.35** | **1.53** |  | 2828 | **1.42** | **1.37** | **1.48** |
| Familial risks were adjusted for age, sex, time period, region of residence, socioeconomic status, and comorbidities. | |  |  |  |  |  |  |  |  |  |  |  |  |  |  |
| Bold type: 95% CI does not include 1.00. O = observed number of cases with family history of kidney failure; SIR = standardized incidence ratio; CI = confidence interval | | | | | | | | | | | | | | | |
