# Supplementary material for: Familial Risks of Kidney Failure in Sweden: A Nationwide Family Study
Source: PLoS One. 2014 Nov 25;9(11):e113353. doi: 10.1371/journal.pone.0113353 (PMC4244139; doi:10.1371/journal.pone.0113353)
Supplement: Table S4 — Familial risk (sibling/offspring) of concordant and discordant kidney failure in males and females, after excluding kidney cancer in parents/offspring. (DOCX) [file pone.0113353.s004.docx]

| **Table S4. Familial risk (sibling/offspring) of concordant and discordant kidney failure in males and females, after excluding kidney cancer in parents/ offspring.** | | | | | | | | | | | | | | | | |
| --- | --- | --- | --- | --- | --- | --- | --- | --- | --- | --- | --- | --- | --- | --- | --- | --- |
|  |  | Males | | | |  | Females | | | |  | All | | | |  |
| Probands with any type of kidney failure | Subtype of kidney failure in offspring/siblings | O | SIR | 95% CI | |  | O | SIR | 95% CI | |  | O | SIR | 95% CI | |  |
| Acute kidney failure | Acute kidney failure | 148 | 1.08 | 0.91 | 1.27 |  | 82 | 1.05 | 0.83 | 1.30 |  | 230 | 1.07 | 0.93 | 1.21 |  |
|  | Chronic kidney failure | 275 | 1.07 | 0.95 | 1.21 |  | 151 | 1.17 | 0.99 | 1.37 |  | 426 | **1.10** | **1.00** | **1.21** |  |
|  | Unspecified kidney failure | 60 | 1.26 | 0.96 | 1.62 |  | 35 | 1.32 | 0.92 | 1.84 |  | 95 | **1.28** | **1.04** | **1.57** |  |
|  | All kidney failure | 483 | **1.09** | **1.00** | **1.20** |  | 268 | **1.14** | **1.01** | **1.29** |  | 751 | **1.11** | **1.03** | **1.19** |  |
|  |  |  |  |  |  |  |  |  |  |  |  |  |  |  |  |  |
| Chronic kidney failure | Acute kidney failure | 196 | 1.15 | 0.99 | 1.32 |  | 125 | **1.25** | **1.04** | **1.49** |  | 321 | **1.18** | **1.06** | **1.32** |  |
|  | Chronic kidney failure | 693 | **2.05** | **1.90** | **2.20** |  | 387 | **1.98** | **1.79** | **2.19** |  | 1080 | **2.02** | **1.90** | **2.15** |  |
|  | Unspecified kidney failure | 102 | **1.59** | **1.30** | **1.93** |  | 64 | **1.76** | **1.36** | **2.25** |  | 166 | **1.65** | **1.41** | **1.93** |  |
|  | All kidney failure | 991 | **1.73** | **1.62** | **1.84** |  | 576 | **1.74** | **1.60** | **1.88** |  | 1567 | **1.73** | **1.65** | **1.82** |  |
|  |  |  |  |  |  |  |  |  |  |  |  |  |  |  |  |  |
| Unspecified kidney failure | Acute kidney failure | 72 | 1.11 | 0.87 | 1.40 |  | 41 | 1.04 | 0.75 | 1.41 |  | 113 | 1.08 | 0.89 | 1.30 |  |
|  | Chronic kidney failure | 166 | **1.28** | **1.10** | **1.49** |  | 96 | **1.28** | **1.04** | **1.56** |  | 262 | **1.28** | **1.13** | **1.45** |  |
|  | Unspecified kidney failure | 33 | 1.23 | 0.85 | 1.73 |  | 21 | 1.41 | 0.87 | 2.16 |  | 54 | 1.30 | 0.97 | 1.69 |  |
|  | All kidney failure | 271 | **1.23** | **1.08** | **1.38** |  | 158 | **1.22** | **1.04** | **1.43** |  | 429 | **1.22** | **1.11** | **1.35** |  |
|  |  |  |  |  |  |  |  |  |  |  |  |  |  |  |  |  |
| All kidney failure | Acute kidney failure | 416 | **1.11** | **1.01** | **1.23** |  | 248 | **1.14** | **1.00** | **1.29** |  | 664 | **1.12** | **1.04** | **1.21** |  |
|  | Chronic kidney failure | 1134 | **1.57** | **1.48** | **1.66** |  | 634 | **1.58** | **1.46** | **1.71** |  | 1768 | **1.57** | **1.50** | **1.65** |  |
|  | Unspecified kidney failure | 195 | **1.41** | **1.22** | **1.62** |  | 120 | **1.55** | **1.28** | **1.85** |  | 315 | **1.46** | **1.30** | **1.63** |  |
|  | All kidney failure | 1745 | **1.41** | **1.35** | **1.48** |  | 1002 | **1.44** | **1.35** | **1.53** |  | 2747 | **1.42** | **1.37** | **1.48** |  |
| Familial risks were adjusted for age, sex, time period, region of residence, socioeconomic status, and comorbidities. | | | | | | | | | | | | | | | |  |
| Bold type: 95% CI does not include 1.00. O = observed number of cases with family history of kidney failure; SIR = standardized incidence ratio; CI = confidence interval | | | | | | | | | | | | | | | |  |
