# Supplementary material for: Familial Risks of Kidney Failure in Sweden: A Nationwide Family Study
Source: PLoS One. 2014 Nov 25;9(11):e113353. doi: 10.1371/journal.pone.0113353 (PMC4244139; doi:10.1371/journal.pone.0113353)
Supplement: Table S5 — Familial risk of concordant and discordant kidney failure in males and females, follow-up 2001–2010. (DOCX) [file pone.0113353.s005.docx]

| **Table S5. Familial risk of concordant and discordant kidney failure in males and females, follow-up 2001-2010** | | | | | | | | | | | | | | | |  |  |  |  |
| --- | --- | --- | --- | --- | --- | --- | --- | --- | --- | --- | --- | --- | --- | --- | --- | --- | --- | --- | --- |
|  |  | Males | | | |  | Females | | | |  | All | | | |  |  |  | |
| Probands with any type of kidney failure | Subtype of kidney failure in offspring/siblings | O | SIR | 95% CI | |  | O | SIR | 95% CI | |  | O | SIR | 95% CI | |  |  |  | |
| Acute kidney failure | Acute kidney failure | 111 | 1.10 | 0.90 | 1.32 |  | 66 | 1.10 | 0.85 | 1.40 |  | 177 | 1.10 | 0.94 | 1.27 |  |  |  | |
|  | Chronic kidney failure | 214 | 1.08 | 0.94 | 1.23 |  | 112 | 1.05 | 0.87 | 1.27 |  | 326 | 1.07 | 0.95 | 1.19 |  |  |  | |
|  | Unspecified kidney failure | 54 | **1.58** | **1.19** | **2.06** |  | 27 | 1.49 | 0.98 | 2.16 |  | 81 | **1.55** | **1.23** | **1.92** |  |  |  | |
|  | All kidney failure | 379 | **1.13** | **1.02** | **1.25** |  | 205 | 1.11 | 0.96 | 1.28 |  | 584 | **1.13** | **1.04** | **1.22** |  |  |  | |
|  |  |  |  |  |  |  |  |  |  |  |  |  |  |  |  |  |  |  | |
| Chronic kidney failure | Acute kidney failure | 158 | 1.15 | 0.98 | 1.34 |  | 104 | **1.39** | **1.14** | **1.69** |  | 262 | **1.24** | **1.09** | **1.39** |  |  |  | |
|  | Chronic kidney failure | 525 | **1.93** | **1.76** | **2.10** |  | 301 | **1.90** | **1.69** | **2.13** |  | 826 | **1.92** | **1.79** | **2.05** |  |  |  | |
|  | Unspecified kidney failure | 68 | **1.42** | **1.10** | **1.80** |  | 38 | **1.52** | **1.07** | **2.08** |  | 106 | **1.45** | **1.19** | **1.76** |  |  |  | |
|  | All kidney failure | 751 | **1.64** | **1.52** | **1.76** |  | 443 | **1.72** | **1.56** | **1.89** |  | 1194 | **1.67** | **1.57** | **1.77** |  |  |  | |
|  |  |  |  |  |  |  |  |  |  |  |  |  |  |  |  |  |  |  | |
| Unspecified kidney failure | Acute kidney failure | 55 | 1.03 | 0.78 | 1.35 |  | 34 | 1.07 | 0.74 | 1.49 |  | 89 | 1.05 | 0.84 | 1.29 |  |  |  | |
|  | Chronic kidney failure | 135 | **1.26** | **1.05** | **1.49** |  | 71 | 1.15 | 0.90 | 1.45 |  | 206 | **1.22** | **1.06** | **1.40** |  |  |  | |
|  | Unspecified kidney failure | 23 | 1.17 | 0.74 | 1.76 |  | 13 | 1.19 | 0.63 | 2.04 |  | 36 | 1.18 | 0.82 | 1.63 |  |  |  | |
|  | All kidney failure | 213 | **1.18** | **1.03** | **1.35** |  | 118 | 1.13 | 0.93 | 1.35 |  | 331 | **1.16** | **1.04** | **1.29** |  |  |  | |
|  |  |  |  |  |  |  |  |  |  |  |  |  |  |  |  |  |  |  | |
| All kidney failure | Acute kidney failure | 324 | 1.11 | 0.99 | 1.24 |  | 204 | **1.23** | **1.06** | **1.41** |  | 528 | **1.15** | **1.06** | **1.25** |  |  |  | |
|  | Chronic kidney failure | 874 | **1.51** | **1.41** | **1.61** |  | 484 | **1.48** | **1.35** | **1.62** |  | 1358 | **1.50** | **1.42** | **1.58** |  |  |  | |
|  | Unspecified kidney failure | 145 | **1.43** | **1.20** | **1.68** |  | 78 | **1.44** | **1.14** | **1.80** |  | 223 | **1.43** | **1.25** | **1.63** |  |  |  | |
|  | All kidney failure | 1343 | **1.38** | **1.31** | **1.46** |  | 766 | **1.40** | **1.30** | **1.50** |  | 2109 | **1.39** | **1.33** | **1.45** |  |  |  | |
| Familial risks were adjusted for age, sex, time period, region of residence, socioeconomic status, and comorbidities. | | | | | | | | | | | | | | | | | | |  |
| Bold type: 95% CI does not include 1.00. O = observed number of cases with family history of kidney failure; SIR = standardized incidence ratio; CI = confidence interval | | | | | | | | | | | | | | | | | | |  |
